# Hematopoietic stem cell regeneration through paracrine regulation of the Wnt5a/Prox1 signaling axis

**DOI:** 10.1172/JCI155914

**Published:** 2022-06-15

**Authors:** Qiqi Lin, Limei Wu, Srinivas Chatla, Fabliha A. Chowdhury, Neha Atale, Jonathan Joseph, Wei Du

**Affiliations:** 1 Division of Hematology and Oncology, University of Pittsburgh School of Medicine, Pittsburgh, Pennsylvania, USA.; 2 University of Pittsburgh Medical Center (UPMC) Hillman Cancer Center, Pittsburgh, Pennsylvania, USA.; 3 Fels Cancer Institute for Personalized Medicine, Lewis Katz School of Medicine at Temple University, Philadelphia, Pennsylvania, USA.; 4 University of Pittsburgh School of Medicine, Pittsburgh, Pennsylvania, USA.

**Keywords:** Cell Biology, Hematology, Hematopoietic stem cells

## Abstract

The crosstalk between the BM microenvironment (niche) and hematopoietic stem cells (HSCs) is critical for HSC regeneration. Here, we show that in mice, deletion of the Fanconi anemia (FA) genes *Fanca* and *Fancc* dampened HSC regeneration through direct effects on HSCs and indirect effects on BM niche cells. FA HSCs showed persistent upregulation of the Wnt target Prox1 in response to total body irradiation (TBI). Accordingly, lineage-specific deletion of *Prox1* improved long-term repopulation of the irradiated FA HSCs. Forced expression of Prox1 in WT HSCs mimicked the defective repopulation phenotype of FA HSCs. WT mice but not FA mice showed significant induction by TBI of BM stromal Wnt5a protein. Mechanistically, FA proteins regulated stromal Wnt5a expression, possibly through modulating the Wnt5a transcription activator Pax2. Wnt5a treatment of irradiated FA mice enhanced HSC regeneration. Conversely, Wnt5a neutralization inhibited HSC regeneration after TBI. Wnt5a secreted by LepR^+^CXCL12^+^ BM stromal cells inhibited **β**-catenin accumulation, thereby repressing *Prox1* transcription in irradiated HSCs. The detrimental effect of deregulated Wnt5a/Prox1 signaling on HSC regeneration was also observed in patients with FA and aged mice. Irradiation induced upregulation of *Prox1* in the HSCs of aged mice, and deletion of *Prox1* in aged HSCs improved HSC regeneration. Treatment of aged mice with Wnt5a enhanced hematopoietic repopulation. Collectively, these findings identified the paracrine Wnt5a/Prox1 signaling axis as a regulator of HSC regeneration under conditions of injury and aging.

## Graphical abstract

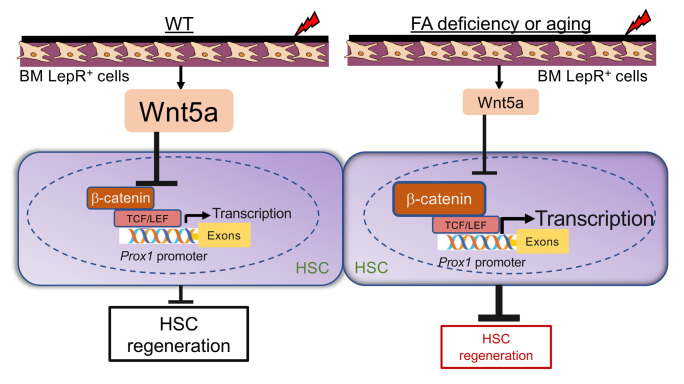

## Introduction

Hematopoietic stem cells (HSCs) in adult humans and mice predominantly reside in the BM, where they preserve the capacity to self-renew and regenerate the whole hematopoietic system (1–3). The BM also contains a variety of nonhematopoietic components, including endosteal and sinusoidal endothelial cells, mesenchymal stromal cells (MSCs), and osteoblast-lineage cells (1, 2, 4, 5). It has long been recognized that nonhematopoietic BM-derived stromal cells are capable of supporting long-term hematopoiesis in vivo and in vitro. For example, studies have shown that maintenance of the HSC pool in mice was dependent upon the expression of stem cell factor by perivascular endothelial cells (6, 7) and that adult sources of endothelial cells produce soluble growth factors that promote the expansion of human HSCs in vitro and support the regeneration of murine and human HSCs in vivo after radiation exposure (8–12). Leptin receptor (LepR) is a marker that is highly enriched in BM MSCs (13). LepR^+^ cells are the main source of new osteoblasts and adipocytes in adult BM and could form bone ossicles that support hematopoiesis in vivo (13). In addition, LepR^+^ perivascular stromal cells have been considered as a major source of stem cell factor and CXCL12 in the BM (6, 7).

Fanconi anemia (FA) is a genetic disorder associated with BM failure and hematologic malignancies, including leukemia (14, 15). HSC failure is considered the root cause of BM failure and leukemia in patients with FA. Although many studies in patients and KO mice have shown that FA deficiency leads to severe defects in both quantity (frequencies and absolute numbers) and quality (such as the ability to reconstitute hematopoiesis) of the HSCs (14, 15), the role of the FA pathway in regulating HSC regeneration under conditions of injury is not well understood.

Signaling by the Wnt pathway is involved in cell proliferation, differentiation, polarity, adhesion, and motility during embryonic morphogenesis to adult tissues (16, 17). Mutations in the genes of the Wnt pathway are one of the major causes of tumorigenesis in different tissues (18). In the hematopoietic system, canonical and noncanonical Wnt signaling have been shown to have discrete effects on hematopoiesis (19). For example, several studies have suggested that activation of canonical Wnt signaling promotes HSC self-renewal (20–23). However, a recent study showed that acute radiation injury increased canonical Wnt pathway activation in HSCs and that treatment with the Wnt inhibitor Dkk1 suppressed Wnt signaling and improved hematopoietic regeneration (1). The noncanonical Wnt signals also elicit contrasting responses in HSCs. On the one hand, Wnt5a in the bone marrow niche is required to regenerate HSCs (24). Wnt5a secreted from stromal cells plays a positive role in the maintenance of HSCs in vitro (25). Wnt5a has also been shown to induce HSC quiescence through inhibition of the canonical Wnt pathway, resulting in an increased ability of the HSCs to reconstitute hematopoiesis (26). More recently, it has been shown that Wnt5a can increase the number of HSCs in samples from patients with Schwachman-Diamond syndrome in surrogate ex vivo assays (27). On the other hand, Wnt5a expression in HSCs increases with aging, causing a shift to noncanonical Wnt signaling and a decline in HSC function in older mice (28).

Wnt signaling has been found to act upstream of homeobox genes, including the Prospero-related homeodomain transcription factor 1 (PROX1; refs. 29, 30), which is highly expressed in hippocampal dentate gyri (31–33) and plays important roles in lens development, lymphangiogenesis, differentiation of certain spinal cord interneurons, and the genesis of hippocampal granule cells (34–38). The promoter of the *Prox1* gene contains 2 functional binding sites for β-catenin–TCF/LEF transcriptional coactivators (29). In the context of hematopoiesis, a study using an in vivo RNAi screen identified Prox1 as a potential antagonist of HSC self-renewal (39). However, the role of Prox1 in HSC maintenance remains largely unknown.

In the current study, we investigated the crosstalk between the BM niche and HSCs in HSC regeneration and showed that BM stromal LepR^+^CXCL12^+^ cells increased production of the noncanonical factor Wnt5a, which depressed the HSC antagonist Prox1 through inhibition of the canonical Wnt signaling and maintained regeneration of irradiated HSCs. In mice that were undergoing aging or with DNA repair deficiency, radiation failed to induce niche Wnt5a, leading to upregulated Prox1 and dampened HSC regeneration. Our study thus identified a paracrine Wnt5a/Prox1 signaling axis in regulating HSC regeneration under conditions of injury and aging.

## Results

### FA deficiency compromises hematologic recovery after irradiation.

**Figure 1:**
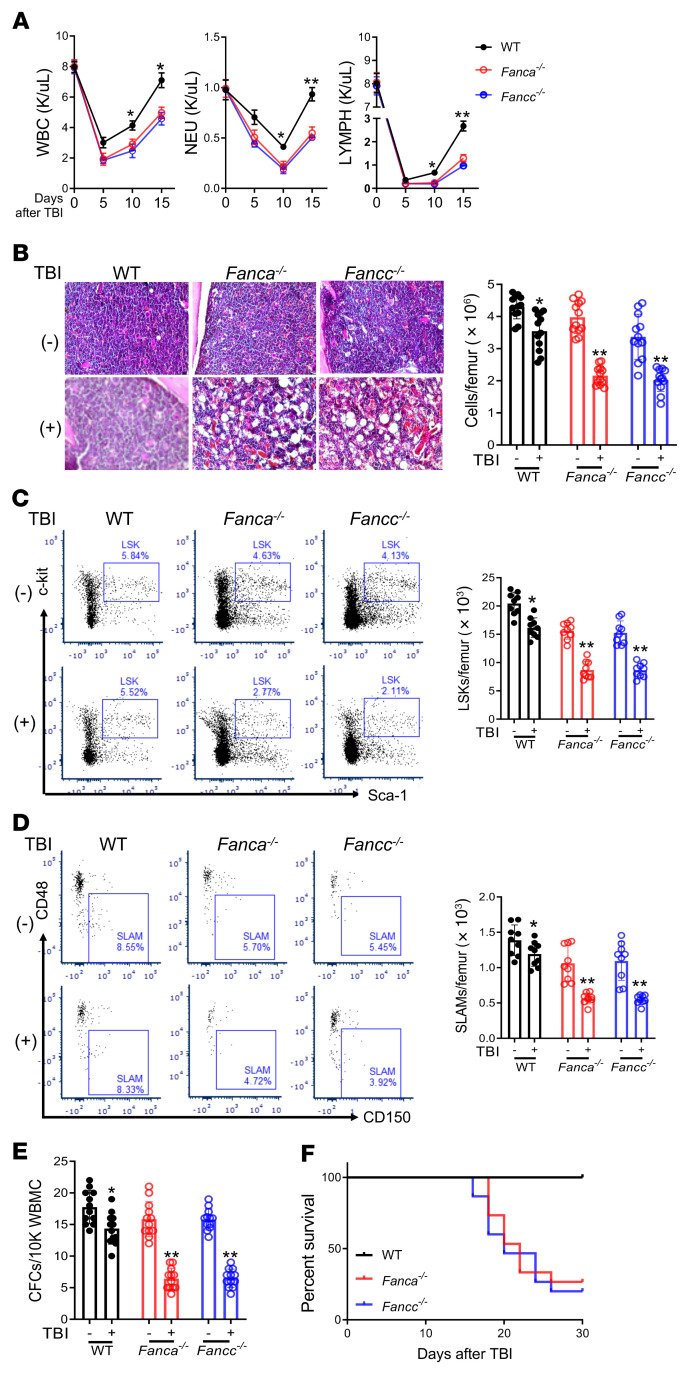
FA deficiency compromises hematologic recovery after irradiation. (**A**) PB WBC, neutrophil (NEU), and lymphocyte (LYMPH) counts in WT, *Fanca^–/–^*, or *Fancc^–/–^* mice at the indicated time points after 500 cGy TBI (*n* = 12/group). (**B**) Left: representative microscopic H&E images (20× original magnification) of BM cellularity in mice described in A on day +15. Right: mean BM cell counts per femur for each group. Results are mean ± SEM of 3 independent experiments (*n* = 12/group). (**C**) Left: representative flow cytometry analysis of percentages of BM LSK (Lin^–^Sca1^+^ckit^+^) cells in the mouse groups shown on day +15. Right: mean numbers of BM LSK cells in each group on day +15 (*n =* 9). (**D**) Left: representative flow cytometry analysis of percentages of BM SLAM (LSKCD48^–^CD150^+^) cells in the mouse groups shown on day +15. Right: mean numbers of BM SLAM cells in each group on day +15 (*n =* 9). (**E**) Mean numbers of BM colony forming cells (CFCs) in the groups shown on day +15 after 500 cGy TBI (*n* = 12). (**F**) Percentage survival of the mouse groups shown through day +30 after 500 cGy TBI (*P <* 0.01 for WT versus *Fanca^–/–^* and *Fancc^–/–^* mice; log-rank test for survival analysis; WT: *n =* 10; *Fanca^–/–^* and *Fancc^–/–^*: *n =* 15). Statistics were performed in the indicated groups: 2-tailed, paired *t* test (parametric). *P < 0.05; **P < 0.01.

To understand the role of the FA pathway in HSC regeneration and hematopoietic recovery in radiation-induced injury, we irradiated *Fanca^–/–^* and *Fancc^–/–^* mice and WT controls with 500 cGy total body irradiation (TBI; ref. 2) and compared the changes in the different hematopoietic contents over time. We found that all the blood parameters, including WBC, neutrophil, and lymphocyte levels in the BM of nonirradiated *Fanca^–/–^* and *Fancc^–/–^* mice were comparable to those in the nonirradiated WT mice (Figure 1A). However, all the blood parameters were significantly lower in *Fanca^–/–^* and *Fancc^–/–^* mice during the 15-day period after TBI than those in the WT control mice (Figure 1A). Consistently, recovery of hemoglobin and hematocrit was also profoundly delayed in the irradiated *Fanca^–/–^* and *Fancc^–/–^* mice compared with that of the irradiated WT controls (Supplemental Figure 1, A–C; supplemental material available online with this article; https://doi.org/10.1172/JCI155914DS1).

We next determined the recovery of BM hematological lineages in mice after 500 cGy TBI and observed a significantly reduced BM cellularity in irradiated *Fanca^–/–^* and *Fancc^–/–^* mice compared with the WT controls on day +15 after TBI as evidenced by H&E staining and total BM cell count (Figure 1B). Flow cytometry analysis revealed a significant decrease in frequencies of LSK (Lin^–^Sca1^+^c-kit^+^; enriched for hematopoietic stem progenitor cells, HSPCs; Figure 1C) and SLAM (LSKCD150^+^CD48^–^; enriched for HSCs; ref. 40) in the irradiated *Fanca^–/–^* and *Fancc^–/–^* mice compared with those in the irradiated WT controls (Figure 1D). In addition, TBI led to a more severe decrease in myeloid and lymphoid lineage cells in *Fanca^–/–^* and *Fancc^–/–^* mice than those in WT mice (Supplemental Figure 1, D–G).

Functionally, colony-forming cells (CFCs) in the irradiated *Fanca^–/–^* and *Fancc^–/–^* mice were markedly reduced compared with WT mice after 500 cGy irradiation (Figure 1E). TBI also caused a profound reduction in survival of *Fanca^–/–^* and *Fancc^–/–^* mice compared with the WT controls (Figure 1F). Taken together, these results indicate that the Fanca and Fancc proteins are required for hematopoietic recovery after irradiation, and suggest that the FA pathway may play an important role in HSC regeneration.

### FA deficiency dampens HSC regeneration after irradiation.

**Figure 2:**
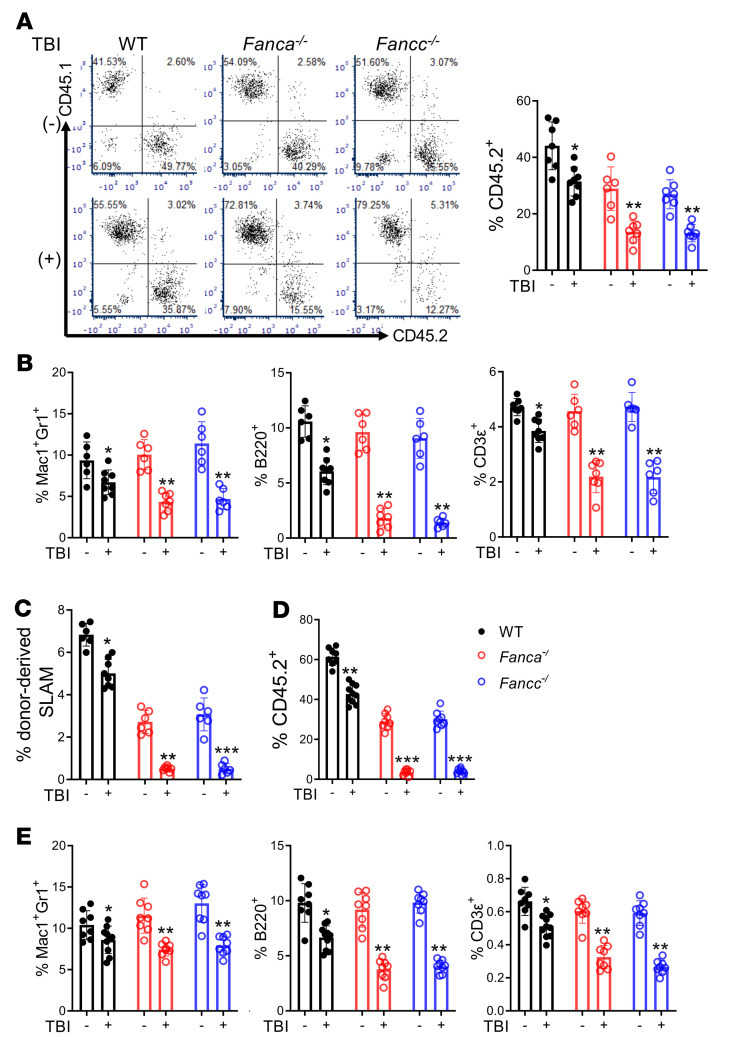
FA deficiency dampens HSC regeneration after irradiation. (**A**) Donor (CD45.2^+^) cell engraftment at 16 weeks in recipient CD45.1^+^ mice that were transplanted with 100 BM SLAM cells from nonirradiated and irradiated WT, *Fanca^–/–^,* and *Fancc^–/–^* mice, along with 2 × 10^5^ competing CD45.1^+^ WT BM cells. Representative flow cytometry analysis (left) and quantification (right) are shown (WT TBI–: *n =* 6; WT TBI+: *n =* 8; *Fanca^–/–^* TBI–: *n =* 6*; Fancc^–/–^* TBI+: *n =* 7*; Fancc^–/–^*: *n =* 6). (**B**) Donor myeloid (Mac1/Gr1), B cell (B220), and T cell (CD3ε) engraftment levels at 16 weeks are shown (WT TBI–: *n =* 6; WT TBI+: *n =* 8; *Fanca^–/–^* TBI–: *n =* 6*; Fancc^–/–^* TBI+: *n =* 7*; Fancc^–/–^*: *n =* 6). (**C**) Donor HSC engraftment at 16 weeks. Mean percentages of CD45.2^+^ SLAM cells are shown for each group (WT TBI–: *n =* 6; WT TBI+: *n =* 8; *Fanca^–/–^* TBI–: *n =* 6*; Fancc^–/–^* TBI+: *n =* 7*; Fancc^–/–^*: *n =* 6). (**D** and **E**) Mean levels of donor CD45.2^+^ cell (**D**) and lineage engraftment (**E**) in secondary recipient CD45.1^+^ mice at 16 weeks after competitive transplantation with BM cells from the primary mice in **A** (WT TBI–: *n =* 8; WT TBI+: *n =* 10; *Fanca^–/–^*: *n =* 8*; Fancc^–/–^*: *n =* 8 in **D**; WT TBI–: *n =* 8; WT TBI+: *n =* 10; *Fanca^–/–^*: *n =* 8*; Fancc^–/–^*: *n =* 8 in **E**). Statistics were performed in the indicated groups: 2-tailed, paired *t* test (parametric). *P < 0.05; **P < 0.01.

To assess the role of the FA pathway in HSC regeneration after radiation, we performed competitive repopulation assays using BM HSCs collected on day +10 from irradiated WT, *Fanca^–/–^*, and *Fancc^–/–^* mice, along with 2 × 10^5^ competitor CD45.1^+^ WT BM cells. We found that primary recipients transplanted with 100 BM SLAM cells from irradiated *Fanca^–/–^* and *Fancc^–/–^* mice displayed significantly lower donor-derived chimerism at 16 weeks after bone marrow transplantation (BMT) than those from the irradiated WT control mice (Figure 2A). The recipient mice transplanted with HSCs from irradiated *Fanca^–/–^* and *Fancc^–/–^* mice showed a significant decrease in multilineage hematopoietic cell reconstitution, as compared with the recipients that were transplanted with HSCs from the irradiated WT mice (Figure 2B). In addition, donor-derived HSCs (CD45.2^+^SLAM), multipotent progenitors (LSKCD48^+^CD150^+^), and hematopoietic progenitor cells (LSKCD48^–^CD150^–^; refs. 40, 41) were also significantly reduced in the recipients transplanted with HSCs from irradiated *Fanca^–/–^* and *Fancc^–/–^* mice (Figure 2C and Supplemental Figure 2, A and B). Donor-derived HSCs from the recipients transplanted with irradiated *Fanca^–/–^* and *Fancc^–/–^* cells were less quiescent compared with those from recipients transplanted with irradiated WT cells (Supplemental Figure 2C). A serial transplantation assay showed that HSCs from irradiated *Fanca^–/–^* and *Fancc^–/–^* mice gave rise to significantly decreased total donor-derived engraftment and multilineage reconstitution compared with WT HSCs in secondary recipient mice (Figure 2, D and E). These data indicate that FA deficiency dampened HSC regeneration after irradiation.

### Prox1 regulates HSC regeneration after irradiation.

**Figure 3:**
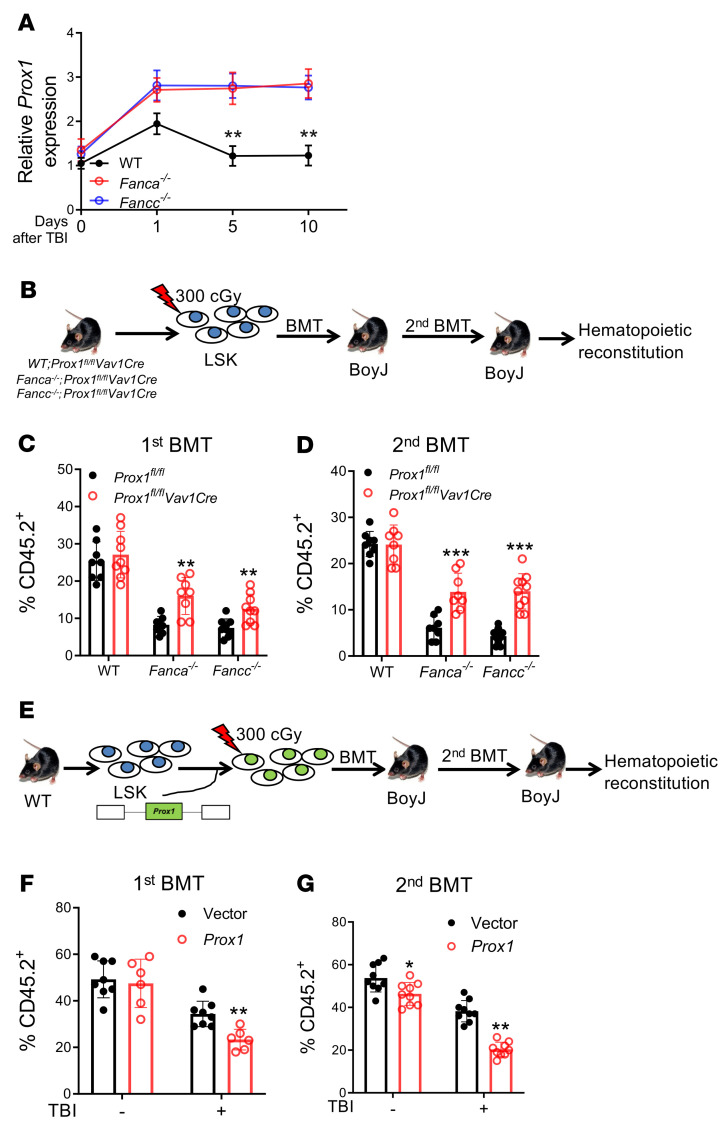
Deletion of *Prox1* improves long-term repopulation of irradiated FA HSCs. (**A**) FA HSCs show persistent upregulation of Prox1 in response to TBI. RNA extracted from SLAM cells isolated from the indicated mice at different time points after 500 cGy TBI was analyzed with qPCR. Samples were normalized to the level of WT *Gapdh* mRNA at day 0 (*n =* 6). (**B**) Schematic diagram of experimental design. (**C**) Deletion of *Prox1* improves repopulation of irradiated FA HSCs. LSK (Lin^–^Sca1^+^c-kit^+^) cells from the indicated mice were subjected to irradiation and transplanted along with competitor cells into BoyJ recipients. Donor-derived chimera were determined at 16 weeks after BMT. *Prox1^fl/fl^:* *n =* 8; *Prox1^fl/fl^Vav1-Cre:* *n =* 9; *Fanca^–/–^;Prox1^fl/fl^:* *n =* 8*; Fanca^–/–^;Prox1^fl/fl^Vav1-Cre:* *n =* 8*; Fancc^–/–^;Prox1^fl/fl^:* *n =* 9*; Fanc9^–/–^;Prox1^fl/fl^Vav1-Cre:* *n =* 9. (**D**) Loss of *Prox1* improves long-term repopulation capacity of FA HSCs. WBMCs from recipients described in **C** were transplanted into BoyJ recipients. *Prox1^fl/fl^:* *n =* 8; *Prox1^fl/fl^Vav1-Cre:* *n =* 9; *Fanca^–/–^;Prox1^fl/fl^:* *n =* 8*; Fanca^–/–^;Prox1^fl/fl^Vav1-Cre:* *n =* 8*; Fancc^–/–^;Prox1^fl/fl^:* *n =* 10*; Fanc9^–/–^;Prox1^fl/fl^Vav1-Cre:* *n =* 10. (**E**) Schematic presentation of experimental design. (**F**) Forced expression of Prox1 mimics FA HSC phenotype in transplanted recipients. LSK cells from WT mice were transduced with lentiviral vector expressing GFP or GFP-*Prox1*. Sorted GFP^+^ cells were subjected to 300 cGy irradiation and transplanted into BoyJ recipients. Vector, TBI–: *n =* 8; vector, TBI+: *Prox1* TBI–: *n =* 7; *Prox1* TBI+: *n =* 6. (**G**) Ectopic overexpression of Prox1 compromises long-term reconstitution of WT HSCs. WBMCs from the recipients described in **F** were transplanted into sublethally irradiated BoyJ recipients (*n =* 9). Statistics were performed in the indicated groups: 2-tailed, paired *t* test (parametric). *P < 0.05; **P < 0.01; ***P < 0.001.

We previously showed that chronic DNA damage caused altered expression of certain DNA damage response (DDR) genes in FA HSPCs (41). To identify the factor(s) responsible for the inhibitory effect on FA HSC regeneration, we screened a panel of DDR genes that were dysregulated by chronic DNA damage in *Fanca^–/–^* and *Fancc^–/–^* mice in our previous transcriptome study (41) and found that *Prox1*, which encodes a homeobox transcription factor, was specifically and persistently upregulated in HSCs and multipotential progenitors from irradiated *Fanca^–/–^* and *Fancc^–/–^* mice (Figure 3A, Supplemental Figure 3A, and Supplemental Figure 3B).

To determine whether Prox1 regulates HSC regeneration, we crossed *Fanca^–/–^* and *Fancc^–/–^* mice with a conditional *Prox1* KO strain (*Prox1^fl/fl^*; ref. 42), in which *Prox1* was specifically deleted in the hematopoietic compartment by a *Vav1-Cre* strain (43). We performed 2 sets of serial BMT assays: first, BM LSK cells from *Fanca^–/–^;Prox1^fl/fl^Vav1-Cre* mice*, Fancc^–/–^;Prox1^fl/fl^Vav1-Cre* mice, and WT control mice were subjected to 300 cGy irradiation followed by serial BMT (Figure 3B). The results showed that ablation of Prox1 significantly increased hematopoietic cell reconstitution by irradiated *Fanca^–/–^* and *Fancc^–/–^* HSCs in both primary and secondary recipient mice compared with mice that were transplanted with irradiated cells from *Fanca^–/–^;Prox1^fl/fl^* and *Fancc^–/–^;Prox1^fl/fl^* control mice (Figure 3, C and D). This improvement in hematopoietic repopulation was correlated with a significant increase in the quiescence of donor-derived HSCs in the recipients transplanted with irradiated cells from *Fanca^–/–^:Prox1^fl/fl^Vav1-Cre* and *Fancc^–/–^;Prox1^fl/fl^Vav1-Cre* mice compared with those in the recipients transplanted with irradiated cells from *Fanca^–/–^;Prox1^fl/fl^* and *Fancc^–/–^;Prox1^fl/fl^* mice (Supplemental Figure 3C). We also noticed that *Prox1* deletion did not affect apoptosis of donor-derived HSCs in the recipients transplanted with irradiated cells from *Fanca^–/–^;Prox1^fl/fl^Vav1-Cre* and *Fancc^–/–^;Prox1^fl/fl^Vav1-Cre* mice (Supplemental Figure 3D). Furthermore, recipient mice transplanted with irradiated cells from *Fanca^–/–^;Prox1^fl/fl^Vav1-Cre* or *Fancc^–/–^;Prox1^fl/fl^Vav1-Cre* mice showed a significant increase in multilineage hematopoietic cell reconstitution in primary and secondary transplanted mice compared with mice transplanted with irradiated cells from *Fanca^–/–^;Prox1^fl/fl^* or *Fancc^–/–^;Prox1^fl/fl^* control mice (Supplemental Figure 3E). It should be noted that deletion of *Prox1* did not affect the hematopoietic repopulating ability of the irradiated WT HSCs (Figure 3, C and D) or the nonirradiated WT or *Fanca^–/–^* and *Fancc^–/–^* HSCs (Supplemental Figure 3F and Supplemental Figure 3G).

In the second set of serial BMT assays, we overexpressed Prox1 in WT LSK cells and subjected the cells to 300 cGy irradiation followed by serial BMT (Figure 3E). We achieved 2-fold higher *Prox1* expression in Prox1-expressing cells than in vector control cells (Supplemental Figures 3, H and I). Remarkably, overexpression of Prox1 profoundly compromised the hematopoietic repopulating ability of the irradiated WT HSCs in primary and secondary transplanted mice compared with the vector-transduced cells (Figure 3, F and G, and Supplemental Figure 3J). Together, these data suggest that Prox1 plays a crucial role in the regulation of HSC regeneration in response to radiation injury.

### Wnt5a from LepR^+^CXCL12^+^ cells regulates hematopoietic recovery and HSC regeneration after irradiation.

**Figure 4:**
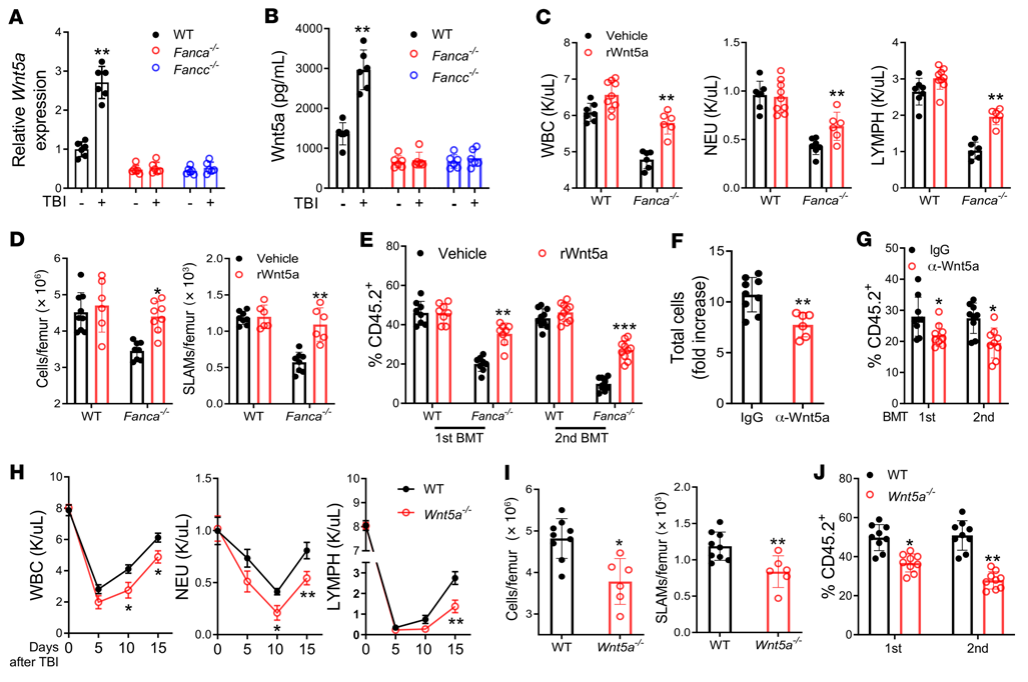
Wnt5a from LepR^+^CXCL12^+^ cells regulates hematopoietic recovery and HSC regeneration after irradiation. (**A**) *Wnt5a* expression in LepR^+^ cells at 24 hours after 500 cGy TBI (*n =* 6). (**B**) Wnt5a protein levels in the BM supernatants of the indicated mice at 24 hours after TBI (*n =* 6). (**C**) Mouse PB parameters at day 22 after TBI. Mice were administered 500 cGy TBI and treated i.p. with rWnt5a (50 μg/kg) or vehicle (saline). WT, vehicle: *n =* 7; WT rWnt5a: *n =* 9; *Fanca^–/–^,* vehicle: *n =* 6; *Fanca^–/–^* rWnt5a: *n =* 6. (**D**) Total BM cells (left) and SLAM cells (right) in mice described in **C**. WT, vehicle: *n =* 7; WT rWnt5a: *n =* 9; *Fanca^–/–^,* vehicle: *n =* 6; *Fanca^–/–^* rWnt5a: *n =* 6. (**E**) First (*n =* 8) and second (*n =* 10) BMT with cells from mice described in **C** (first, 8; second, 10). (**F**) Effect of anti-Wnt5a neutralization on HSC expansion. WT SLAM cells were subjected to 300 cGy irradiation and cocultured with WT LepR^+^ cells and anti-Wnt5a (2 μg/mL) or control IgG for 5 days (IgG: *n =* 9; α-Wnt5a: *n =* 6). (**G**) Neutralization of Wnt5a dampens the repopulation capacity of the irradiated WT HSCs. 1000 progeny cells from the cocultures described in **F** were subjected to serial BMT (*n =* 9). (**H**) Deletion of *Wnt5a* delays PB recovery after irradiation. WT: *n =* 9; *Wnt5a^–/–^,* 0: *n =* 7; *Wnt5a^–/–^,* 5, 10, 15: *n =* 6. (**I**) Mean total BM cells (left) and SLAM cells (right) for the mice described in **H** at day 15 after TBI. WT: *n =* 9; *Wnt5a^–/–^*: *n =* 6. (**J**) Deletion of *Wnt5a* dampens HSC repopulation after irradiation. BM SLAM cells from the mice described in **H** were subjected to BMT (*n =* 9). Statistics were performed in the indicated groups: 2-tailed, paired *t* test (parametric). *P < 0.05; **P < 0.01.

Previous studies have established Prox1 as a target of Wnt signaling (29, 44, 45). To identify the Wnt factors that regulate *Prox1* expression, we performed Wnt pathway–specific gene profiling in HSCs and 3 major BM niche components (CD45^–^LepR^+^, CD45^–^Osx^+^, and CD45^–^VE-cad^+^ cells) from nonirradiated mice and irradiated mice at 48 hours after 500 cGy TBI using QIAGEN RT^2^ profiler PCR arrays. We found that BM stromal CD45^–^LepR^+^ cells from WT mice, but not those from *Fanca^–/–^* mice, exhibited irradiation-responsive *Wnt5a* upregulation (Supplemental Figure 4A). In addition, this TBI–induced *Wnt5a* upregulation was not observed in HSCs or the other 2 BM niche cells (CD45^–^Osx^+^ and CD45^–^VE-cad^+^; Supplemental Figure 4A and Supplemental Figure 4B). We performed qPCR assays and confirmed the differential and irradiation-responsive *Wnt5a* upregulation in WT and FA BM stromal CD45^–^LepR^+^ cells (Figure 4A). Furthermore, analysis for the Wnt5a protein by both ELISA assay and flow cytometry–based intracellular staining showed a significant induction of BM stromal Wnt5a protein in CD45^–^LepR^+^ cells from WT mice, but not those from FA mice, in response to irradiation (Figure 4B and Supplemental Figure 4C). Further analysis showed that irradiation induced Wnt5a specifically in BM LepR^+^CXCL12^+^ stromal cells (Supplemental Figure 4D). These results indicate that BM LepR^+^CXCL12^+^ stromal cells were the primary source of Wnt5a that is differentially responsive to irradiation.

Because *Fanca^–/–^* and *Fancc^–/–^* mice failed to induce stromal Wnt5a in response to irradiation and showed dampened hematopoietic recovery after TBI, we hypothesized that irradiation-induced stromal Wnt5a might promote hematopoietic regeneration. We thus sought to determine whether Wnt5a regulates hematopoietic regeneration in response to irradiation. We first evaluated the effect of Wnt5a on the function of nonirradiated HSCs in vitro and in vivo. Treatment of nonirradiated BM SLAM cells from WT, *Fanca^–/–^*, and *Fancc^–/–^* mice with recombinant Wnt5a (rWnt5a; 100 ng/mL; ref. 28) in cytokine-supplemented medium had no effect on total BM cell or SLAM cell expansion of WT cells compared with cells treated with vehicle (saline) (Supplemental Figure 5A). Wnt5a increased expansion of *Fanca^–/–^* and *Fancc^–/–^* SLAM, although it was not statistically significant (Supplemental Figure 5A). Next, we assessed the in vivo effect of rWnt5a on nonirradiated mice. Systemic treatment with rWnt5a (50 μg/kg; i.p.; ref. 46) every other day for 3 weeks resulted in an approximate 6-fold increase in rWnt5a levels in the BM of WT, *Fanca^–/–^*, and *Fancc^–/–^* mice at 1 hour after treatment compared with vehicle-treated mice (Supplemental Figure 5B). rWnt5a treatment did not affect the numbers of BM total cells or BM SLAM cells of WT mice; however, systemic administration of Wnt5a caused a significant increase in the numbers of BM SLAM cells of *Fanca^–/–^* and *Fancc^–/–^* mice compared with saline-treated mice (Supplemental Figure 5C). These results indicate that Wnt5a promoted the in vivo expansion of FA HSCs under steady state.

We next assessed the function of Wnt5a in regulating hematopoietic regeneration after irradiation. WT, *Fanca^–/–^*, and *Fancc^–/–^* mice were irradiated with 500 cGy TBI and treated with rWnt5a (50 μg/kg; i.p.) or vehicle (saline) every other day for 3 weeks. Systemic treatment of rWnt5a enhanced recovery in peripheral blood (PB) WBCs, neutrophils, and lymphocytes of *Fanca^–/–^* and *Fancc^–/–^* mice after irradiation compared with vehicle-treated control mice (Figure 4C and Supplemental Figure 5D). This enhanced hematopoietic recovery by Wnt5a treatment was associated with improved total BM cell and SLAM cell counts in FA mice (Figure 4D and Supplemental Figure 5E). Furthermore, serial BMT assays with LSK cells from the mice subjected to the above systemic treatments showed that Wnt5a treatment greatly improved hematopoietic repopulation of *Fanca^–/–^* and *Fancc^–/–^* HSCs in both primary and secondary recipients (Figure 4E and Supplemental Figure 5F). It appears that the improved hematopoietic repopulation was at least in part through enhancing HSC quiescence (Supplemental Figure 5G), not via reducing apoptosis (Supplemental Figure 5H). Thus, systemic administration of Wnt5a enhanced hematopoietic regeneration in vivo after TBI.

We then tested whether inhibition of Wnt5a signaling compromises HSC regeneration after irradiation. We took 2 approaches: a) pharmacological neutralization of Wnt5a using anti-Wnt5a antibody; b) genetic *Wnt5a* KO in the BM niche using a previously described *Wnt5a^fl/fl^* mouse model (47) crossed with a *LepR-Cre* deleter strain (48). We first cocultured WT SLAM cells with BM LepR^+^ cells isolated from WT mice in the presence of anti-Wnt5a antibody (49) or control IgG for 5 days after 300 cGy irradiation. We observed a significant reduction of total progenies of the coculture in the presence of anti-Wnt5a antibody compared with that of the IgG control (Figure 4F). Serial BMT assays further demonstrated that neutralization of Wnt5a dampened the repopulation capacity of the irradiated WT HSCs in both primary and secondary recipients (Figure 4G). Genetic deletion of *Wnt5a* showed a delayed PB recovery over time, as evidenced by reduced WBC, neutrophil, and lymphocyte counts in *Wnt5a^–/–^* (*Wnt5a^fl/fl^;LepR-Cre*) mice at days 10 and 15 after TBI (Figure 4H). Furthermore, *Wnt5a* KO significantly decreased total BM cells and BM SLAM cells at day 15 after TBI compared with the WT control (Figure 4I). Serial BMT assays showed that *Wnt5a* deletion in the BM niche profoundly impaired hematopoietic repopulation of the HSCs in both primary and secondary recipients (Figure 4J). However, deletion of *Wnt5a* in *Fanca^–/–^* and *Fancc^–/–^* mice did not further reduce PB cell counts and total BM cells or SLAM cells, or hematopoietic repopulation in both primary and secondary recipients (Supplemental Figure 5I–5K). Therefore, these data demonstrated that Wnt5a improved hematopoietic recovery and HSC regeneration after irradiation.

### LepR^+^ niche cell–derived Wnt5a inhibits β-catenin accumulation and represses Prox1 expression in irradiated HSPCs.

**Figure 5:**
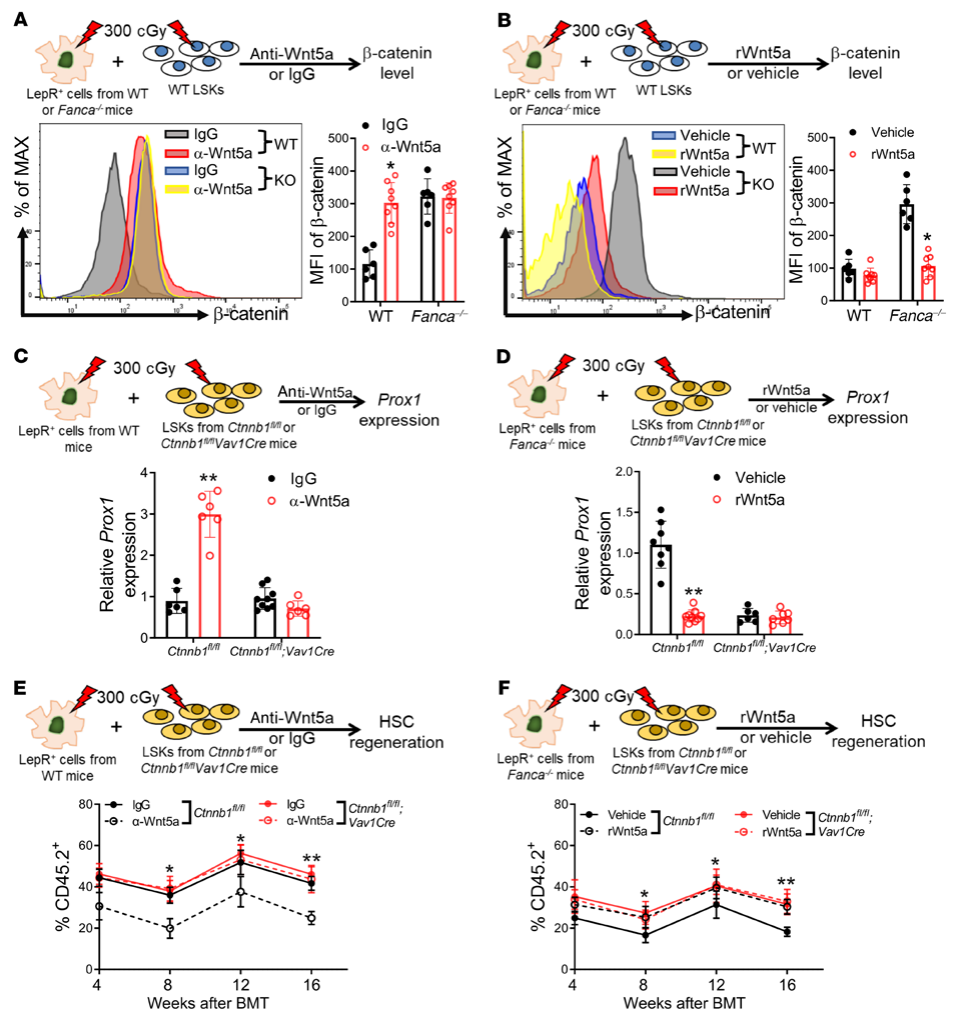
LepR^+^ niche cell–derived Wnt5a inhibits β-catenin accumulation and represses *Prox1* expression in irradiated HSPCs. (**A**) Wnt5a neutralization increases β-catenin accumulation in cocultured HSPCs. LSK cells and LepR^+^ cells were subjected to 300 cGy irradiation and cocultured with anti-Wnt5a (2 μg/mL) or control IgG for 5 days. Levels of β-catenin in suspension cells were determined. IgG: *n =* 6; α-Wnt5a: *n =* 8. (**B**) Wnt5a treatment reduces β-catenin accumulation in cocultured HSPCs. WT LSK cells and LepR^+^ cells were subjected to 300 cGy irradiation and cocultured with rWnt5a (100 ng/mL) or vehicle (saline) for 5 days. IgG: *n =* 6; α-Wnt5a: *n =* 8. (**C**) Deletion of *Ctnnb1* abrogates the effect of Wnt5a neutralization on *Prox1* expression in cocultured HSPCs. LSK cells from *Ctnnb1^fl/fl^* or *Ctnnb1^fl/fl^Vav1-Cre* mice were cocultured with WT LepR^+^ cells and anti-Wnt5a (2 μg/mL) or control IgG for 5 days after 300 cGy irradiation. *Prox1* expression was determined. *Ctnnb1^fl/fl^Vav1-Cre,* IgG: *n =* 9; others: *n =* 6. (**D**) Deletion of *Ctnnb1* abolishes the effect of rWnt5a. LSK cells were cocultured with LepR^+^ cells in the presence of rWnt5a (100 ng/mL) or vehicle for 5 days after 300 cGy irradiation. *Ctnnb1^fl/fl^*, vehicle: *n =* 8; *Ctnnb1^fl/fl^*, rWnt5a: *n =* 9; *Ctnnb1^fl/fl^Vav1-Cre,* vehicle: *n =* 6; *Ctnnb1^fl/fl^Vav1-Cre,* rWnt5a: *n =* 7. (**E**) *Ctnbb1* deletion abrogates the dampening effect of Wnt5a neutralization to 1000 progeny cells from cocultures were transplanted into BoyJ recipients. *Ctnnb1^fl/fl^Vav1-Cre,* α-Wnt5a: *n =* 12; others, *n* = 10. (**F**) *Ctnbb1* deletion abolishes the promoting effect of rWnt5a to 1000 progeny cells from cocultures of were transplanted into BoyJ recipients. *Ctnnb1^fl/fl^*, vehicle; *Ctnnb1^fl/fl^Vav1-Cre,* rWnt5a: *n =* 10; *Ctnnb1^fl/fl^*, rWnt5a; *Ctnnb1^fl/fl^Vav1-Cre,* vehicle: *n =* 12. Statistics were performed in the indicated groups: 2-tailed, paired *t* test (parametric). *P < 0.05; **P < 0.01.

Previous work has shown that Wnt5a inhibits β-catenin accumulation (16, 17). Since β-catenin is a coactivator of *Prox1* transcription (45), we hypothesized that irradiation-induced niche Wnt5a might act to reduce β-catenin, thereby repressing *Prox1* expression in HSCs. To test this, we cocultured WT LSK cells with LepR^+^ cells isolated from WT mice in the presence of anti-Wnt5a antibody or control IgG for 5 days after 300 cGy irradiation and measured β-catenin accumulation in the suspension cells. We found that neutralization of Wnt5a by anti-Wnt5a antibody led to a significant increase in β-catenin accumulation in WT LSKs cocultured on WT LepR^+^ cells after irradiation (Figure 5A). Conversely, addition of rWnt5a reduced β-catenin accumulation in WT LSKs cocultured on LepR^+^ cells from *Fanca^–/–^* and *Fancc^–/–^* mice after irradiation (Figure 5B and Supplemental Figure 6A).

To further establish the mechanistic link between irradiation-induced niche Wnt5a and HSC *Prox1* expression, we ablated the *Prox1* transcription activator β-catenin specifically in hematopoietic lineages by employing a previously described conditional *Ctnnb1* KO mouse model (*Ctnnb1^fl/fl^*; ref. 50) and the *Vav1-Cre* mice (43). We found that Wnt5a neutralization caused a significant increase of *Prox1* mRNA level in cocultured *Ctnnb1^fl/fl^* (*Ctnnb1*-undeleted*)* LSK cells compared with the IgG control (Figure 5C). In contrast, anti-Wnt5a antibody treatment failed to increase *Prox1* expression in cocultured *Ctnnb1^fl/fl^Vav1-Cre* LSK cells (Figure 5C). Furthermore, addition of rWnt5a led to a significant decrease in *Prox1* expression in the cocultured cells from *Ctnnb1^fl/fl^* mice, but not those from *Ctnnb1^fl/fl^Vav1-Cre* mice, on *Fanca^–/–^* or *Fancc^–/–^* LepR^+^ cells (Figure 5D and Supplemental Figure 6B). These results suggest that the irradiation-induced niche Wnt5a repressed *Prox1* expression in cocultured HSPCs through inhibition of β-catenin.

To determine the functional relevance of *Prox1* repression by β-catenin inhibition to Wnt5a-mediated hematopoietic regeneration, we performed BMT using the progenies from the cocultures of WT LepR^+^ cells plus *Ctnbb1^fl/fl^Vav1-Cre* LSK plus anti-Wnt5a or those from the cocultures of FA LepR^+^ cells plus *Ctnbb1^fl/fl^Vav1-Cre* LSK plus rWnt5a. We found that in the WT LepR^+^ cells with *Ctnbb1^fl/fl^Vav1-Cre* LSK coculture, ablation of β-catenin by *Ctnbb1* deletion rendered the cocultured HSPCs insensitive to the dampening effect of Wnt5a neutralization on hematopoietic regeneration (Figure 5E). In the FA LepR^+^ cells with *Ctnbb1^fl/fl^Vav1-Cre* LSK coculture, addition of rWnt5a significantly improved hematopoietic regeneration of the cocultured *Ctnnb1^fl/fl^* (*Ctnnb1*-undeleted) LSK cells compared with IgG control (vehicle) (Figure 5F and Supplemental Figure 6C). However, addition of rWnt5a did not further increase hematopoietic regeneration of the cocultured *Ctnbb1^fl/fl^Vav1-Cre* LSK cells (Figure 5F and Supplemental Figure 6C). Taken together, these data suggest that LepR^+^ niche cell–derived Wnt5a promotes hematopoietic regeneration after irradiation via inhibition of β-catenin accumulation, thereby repressing *Prox1* expression in HSCs.

### Altering Wnt5a/Prox1 signaling compromises HSC regeneration and hematopoietic recovery in aged mice.

It is well known that aging is associated with declined HSC regeneration (51, 52). We hypothesized that Wnt5a/Prox1 signaling might be altered during aging, which would lead to compromised HSC regeneration. We first asked if *Prox1* expression was upregulated in the HSCs of aged mice in response to TBI. Indeed, we found that the level of *Prox1* expression was higher in old HSCs than in young HSCs in steady state and was further elevated in response to irradiation (Supplemental Figure 7A). Old mice exhibited prolonged delay in hematopoietic recovery after irradiation, as evidenced by significantly lower WBC, neutrophil, and lymphocyte counts in irradiated old mice than in young mice at day 10 and day 15 after irradiation (Supplemental Figure 7B). Old mice also showed significantly slower recovery than young mice in the HSPC (LSK) compartment after irradiation (Supplemental Figure 7C).

**Figure 6:**
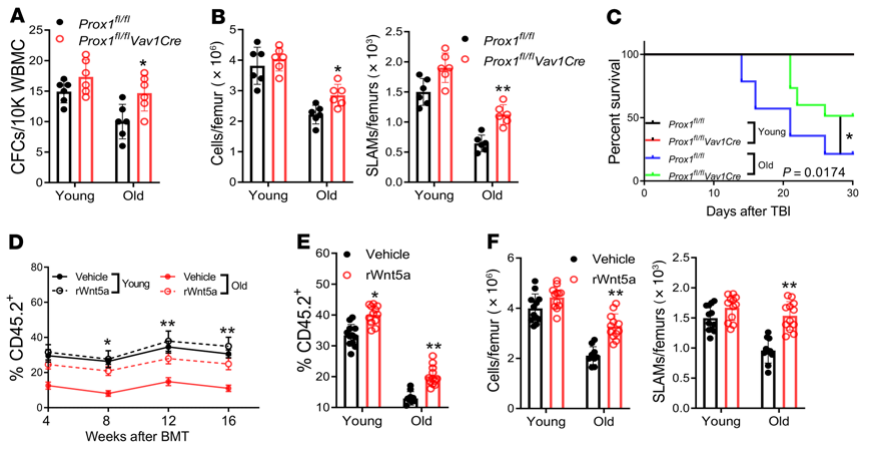
Effect of Wnt5a/Prox1 signaling on HSC regeneration and hematopoietic recovery in aged mice. (**A**) Deletion of *Prox1* improves CFC recovery in aged mice after irradiation. Mean numbers of BM CFCs in nonirradiated and irradiated (15 days after 500 cGy TBI) young and old *Prox1^fl/fl^* or *Prox1^fl/fl^Vav1-Cre* mice (*n =* 6). (**B**) Deletion of *Prox1* improves BM and HSC recovery in aged mice after irradiation. Mean numbers of total BM cells (left) and BM SLAM cells (right) in nonirradiated and radiated young and old mice (*n =* 6). (**C**) Ablation of Prox1 increases survival of irradiated aged mice (young *Prox1^fl/fl^:* *n =* 15; young *Prox1^fl/fl^Vav1-Cre: n =* 15; old *Prox1^fl/fl^:* *n =* 14; old *Prox1^fl/fl^Vav1-Cre: n =* 15). Old *Prox1^fl/fl^* versus *Prox1^fl/fl^Vav1-Cre* mice: *P =* 0.0174. (**D**) Wnt5a improves regeneration of aged HSCs after irradiation. SLAM cells from young and old mice were irradiated at 300 cGy and cultured in the presence of rWnt5a (100 ng/mL) or vehicle (saline) for 5 days. 500 progeny cells from the cultures were transplanted into BoyJ recipients (*n =* 12). (**E**) Mean levels of donor CD45.2^+^ cell engraftment in secondary recipients at 16 weeks following transplantation with BM cells from the primary mice in **D** (old vehicle: *n =* 10; others: *n =* 12). (**F**) Systemic administration of rWnt5a improves hematopoietic recovery in aged mice after irradiation. Total BM cells (left) and SLAM cells (right) in young and old mice on day 22 after 500 cGy TBI. Mice were subjected to 500 cGy TBI and treated i.p. with rWnt5a (50 μg/kg) or vehicle (saline) (old vehicle: *n* = 10; others: *n* = 12). Statistics were performed in the indicated groups: 2-tailed, paired *t* test (parametric). *P < 0.05; **P < 0.01.

We next tested whether deletion of *Prox1* would improve HSC regeneration in aged mice. By employing a hematopoietic lineage–specific *Prox1* KO mouse model (*Prox1^fl/fl^ Vav1-Cre)*, we found that ablation of Prox1 improved CFC recovery in aged mice after irradiation (Figure 6A). Significantly, the numbers of total BM cells and BM SLAM cells were also increased in aged *Prox1^fl/fl^ Vav1-Cre* mice after irradiation compared with those in the control (*Prox1^fl/fl^*) mice (Figure 6B). Furthermore, ablation of Prox1 significantly increased the 30-day survival of irradiated old mice compared with irradiated controls (Figure 6C). These results suggest that Prox1 plays an important role in regulating hematopoietic regeneration and survival in aged mice after irradiation.

Nonirradiated and irradiated old mice showed a significantly lower level of the Wnt5a protein in the BM supernatants than those of the young control mice (Supplemental Figure 7D). To investigate the link between altered Wnt5a production and compromised hematopoietic regeneration in aged mice, we subjected SLAM cells from young and old mice to 300 cGy irradiation followed by culture in the presence of rWnt5a or vehicle for 5 days. The progenies of these cultures were used for serial BMT assays. We observed significantly increased hematopoietic reconstitution in both primary and secondary recipients transplanted with the progenies of rWnt5a-treated SLAM cells from old mice compared with those treated with vehicle control (Figure 6, D and E). It should be noted that the progenies of young SLAM cells treated with rWnt5a also gave rise to higher hematopoietic repopulation in the secondary recipients compared with those treated with vehicle (Figure 6E). The recipient mice transplanted with the progenies of rWnt5a-treated SLAM cells from old mice showed a significant increase in multilineage hematopoietic cell reconstitution compared with the mice transplanted with those of vehicle-treated aged SLAM cells (Supplemental Figure 7E). Furthermore, systemic administration of rWnt5a to the irradiated old mice significantly increased the numbers of total BM cells and BM SLAM cells compared with the irradiated old mice treated with vehicle (Figure 6F). Taken together, these results indicate that altering Wnt5a/Prox1 signaling compromised HSC regeneration and hematopoietic recovery in aged mice.

### Dysregulated paracrine WNT5a/PROX1 axis in patients with FA.

**Figure 7:**
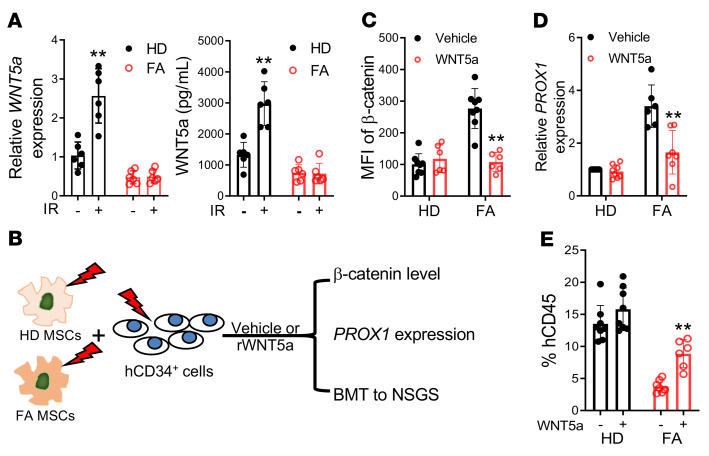
Dysregulated paracrine WNT5a/PROX1 axis in patients with FA. (**A**) MSCs from healthy donors (HDs) but not patients with FA show significant induction of Wnt5a in response to irradiation. MSCs from HDs or patients with FA were cultured in MSC culture medium. The levels of mRNA (left) and protein (right) of WNT5a were measured by qPCR and ELISA, respectively (*n =* 6). (**B**) Schematic presentation of experimental design. Healthy BM hCD34^+^ cells cocultured with irradiated MSCs from HDs or patients with FA followed by β-catenin staining, qPCR analysis, or BMT. (**C**) Recombinant WNT5a reduces β-catenin accumulation in cocultured human HSPCs. Healthy hCD34^+^ cells and MSCs from HDs were subjected to 300 cGy irradiation and then cocultured for 5 days in the presence of rWNT5a or vehicle control; β-catenin levels were determined in the suspension cells by flow cytometry analysis. MFI of β-catenin shown (HD vehicle: *n =* 8; HD WNT5a: *n =* 6; FA vehicle: *n =* 8; FA WNT5a: *n =* 6). (**D**) rWNT5a represses *PROX1* expression in hCD34^+^ cells cocultured on FA MSCs. Healthy hCD34^+^ cells and MSCs cells from HDs or patients with FA were subjected to 300 cGy irradiation followed by coculture for 5 days in the presence of recombinant WNT5a or vehicle control. Suspension cells were collected for RNA extract and qPCR analysis for *PROX1* expression (HD vehicle: *n =* 8; HD WNT5a: *n =* 8; FA vehicle: *n =* 6; FA WNT5a: *n =* 7). (**E**) rWNT5a improves repopulating capacity of the progenies of hCD34^+^ cells cocultured on FA MSC in NSGS recipients. Ten thousand progeny cells after coculture in the presence of recombinant WNT5a or vehicle control for 5 days were transplanted into sublethally irradiated NSGS mice. Human engraftment at 16 weeks after BMT were determined by flow cytometry. Statistics were performed in the indicated groups: 2-tailed, paired *t* test (parametric). *P < 0.05; **P < 0.01.

To evaluate whether our findings in the mouse models were extendable to humans, we assessed irradiation-induced WNT5a expression in BM-derived MSCs from patients with FA. We found that irradiation induced a significant increase in WNT5a expression in MSCs derived from healthy donors compared with those from patients with FA, as detected by both qPCR analysis and ELISA assay (Figure 7A). We then performed MSC-human CD34^+^ cell coculture experiments (Figure 7B) and found that coculture of healthy donor CD34^+^ cells with FA MSCs resulted in high levels of β-catenin accumulation (Figure 7C) and increased *PROX1* expression (Figure 7D) compared with those cocultured with healthy donor MSCs. Addition of rWNT5a reduced β-catenin accumulation (Figure 7C) and *PROX1* expression (Figure 7D) in human CD34^+^ cells cocultured on FA MSCs after irradiation. Functionally, the progenies of hCD34^+^ cells from the cocultures with FA MSCs gave rise to significantly lower human engraftment in NSG-SGM3 (NSGS) recipients (Figure 7E). rWNT5a treatment significantly improved the repopulating capacity of the progenies of hCD34^+^ cells cocultured with FA MSCs (Figure 7E). These results suggest that the impairment of the irradiation-responsive Wnt5a/Prox1 signaling axis may play a causal role in the defect of HSCs from patients with FA.

### FA deficiency reduces stromal Wnt5a via downregulation of Wnt5a transcription activators.

**Figure 8:**
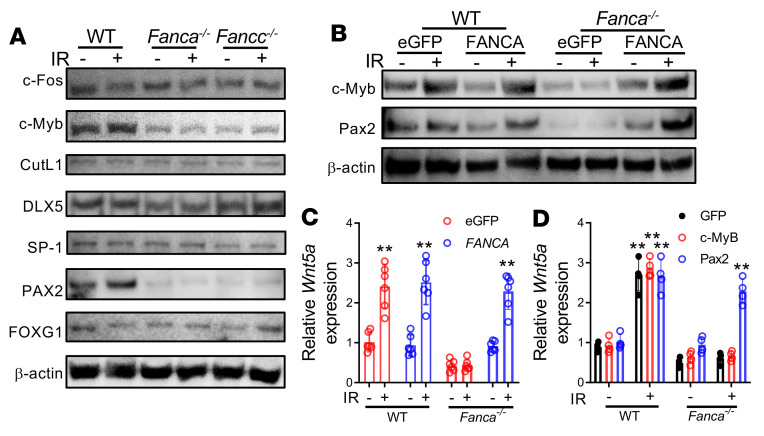
FA deficiency reduces stromal Wnt5a via downregulation of *Wnt5a* transcription activators. (**A**) Downregulation of *Wnt5a* transcription activators c-Myb and Pax2 in FA MSCs. Western blotting of known *Wnt5a* transcription activators and repressors in whole cell lysates (WCLs) extracted from irradiated or control WT, *Fanca^–/–^,* and *Fancc^–/–^* MSCs using the indicated antibodies. (**B**) Genetic correction of FA deficiency restores both steady-state and irradiation-responsive levels of c-Myb and Pax2. MSCs from WT or *Fanca^–/–^* mice were transduced with retroviral vectors expressing eGFP-FANCA or eGFP alone, and the sorted GFP^+^ cells were subjected to irradiation (300 cGy). WCL from irradiated or control MSCs were subjected to immunoblotting using antibodies against c-Myb, Pax2, or β-actin. (**C**) Genetic correction of FA deficiency rescues both steady-state and irradiation-induced Wnt5a expression in FA MSCs. The MSCs described in **B** were subjected to qPCR analysis for *Wnt5a* expression. Statistics were performed in the indicated groups: 2-tailed, paired *t* test (parametric). **P < 0.01. WT + eGFP IR (–) versus WT + eGFP IR (+): *P =* 0.0035; WT + FANCA IR (–) versus WT + FANCA IR (+): *P =* 0.0030; *Fanca^–/–^* + FANCA IR (–) versus *Fanca^–/–^* + FANCA IR (+): *P =* 0.0010. (**D**) Forced expression of Pax2 restores *Wnt5a* expression in *Fanca^–/–^* MSCs. MSCs from WT and *Fanca^–/–^* mice were transduced with retroviral vectors expressing eGFP, c-Myb, or Pax2. Sorted GFP^+^ cells were subjected to qPCR analysis for *Wnt5a* expression. Statistics were performed in the indicated groups: 2-tailed, paired *t* test (parametric). WT + eGFP IR (–) versus WT + eGFP IR (+): *P =* 0.0057; WT + c-MyB IR (–) versus WT + c-MyB IR (+): *P =* 0.0020; WT + Pax2 IR (–) versus WT + Pax2 IR (+): *P =* 0.0016; *Fanca^–/–^* + Pax2 IR (–) versus *Fanca^–/–^* + Pax2 IR (+): *P =* 0.0063.

The observation that the levels of Wnt5a mRNA and protein in BM stromal CD45^–^LepR^+^ cells from *Fanca^–/–^* and *Fancc^–/–^* mice were significantly lower than those from WT mice (Figure 4A) prompted us to postulate that FA proteins regulate the production of stromal Wnt5a, which acts in a paracrine manner to modulate *Prox1* expression in HSCs. To investigate the mechanistic link between FA deficiency and reduced Wnt5a expression, we first determined whether FA deficiency directly affected stromal Wnt5a transcription. We examined several known *Wnt5a* transcription activators and repressors (53–59) in MSCs from WT, *Fanca^–/–^*, and *Fancc^–/–^* mice and found that the levels of 2 well-known *Wnt5a* transcription activators, c-Myb and Pax2 (53–55), were significantly lower in *Fanca^–/–^* and *Fancc^–/–^* MSCs than in WT MSCs (Figure 8A). Irradiation induced even higher levels of c-Myb and Pax2 in WT stromal cells; however, this responsiveness was not observed in MSCs from *Fanca^–/–^* and *Fancc^–/–^* mice (Figure 8A). Forced expression of *FANCA* or *FANCC* in MSCs from *Fanca^–/–^* or *Fancc^–/–^* mice (Supplemental Figure 8A) restored both steady-state and radiation-responsive levels of c-Myb and Pax2 (Figure 8B and Supplemental Figure 8B). Furthermore, genetic correction of FA deficiency rescued both steady-state and radiation-induced *Wnt5a* expression in *Fanca^–/–^* and *Fancc^–/–^* MSCs (Figure 8C and Supplemental Figure 8C). Interestingly, forced expression of Pax2 but not c-Myb restored *Wnt5a* expression in *Fanca^–/–^* and *Fancc^–/–^* MSCs (Figure 8D and Supplemental Figure 8, D and E). Together, these data suggest that the FA proteins regulate stromal *Wnt5a* expression through modulating transcription activator Pax2.

## Discussion

The current study investigates the crosstalk between the BM microenvironment and HSCs in HSC regeneration after radiation injury using mouse models of DNA repair deficiency (FA) and aging. Our study identified a paracrine Wnt5a/Prox1 signaling axis that regulates HSCs regeneration under conditions of injury and aging. Several lines of evidence highlight our findings: a) FA deficiency dampened HSC regeneration through direct effects on HSCs and indirect effects on BM niche cells; b) FA HSCs showed persistent upregulation of the Wnt target Prox1 in response to TBI; c) deletion of *Prox1* improved long-term repopulation of the irradiated FA HSCs, and forced expression of Prox1 in WT HSCs mimicked the defective repopulation phenotype of FA HSCs; d) TBI significantly induced BM stromal Wnt5a expression in WT mice but not FA mice, which was specifically produced in LepR^+^CXCL12^+^ BM stromal cells; e) Wnt5a treatment of irradiated FA mice enhanced hematopoietic recovery and HSC regeneration; f) Wnt5a secreted from LepR^+^CXCL12^+^ BM stromal cells inhibited β-catenin accumulation, thereby repressing *Prox1* transcription in the irradiated HSPCs; g) the detrimental effect of dysregulated Wnt5a/Prox1 signaling on HSC regeneration and hematopoietic recovery was also observed in aged mice and patients with FA; h) mechanistically, FA proteins regulate *Wnt5a* expression through modulating transcription activator Pax2.

One interesting finding of the present study is that in mice, deletion of genes encoding the DNA repair deficiency syndrome FA, *Fanca* and *Fancc,* dampened HSC regeneration through direct effects on HSCs and indirect effects on BM niche cells. FA is a genomic instability syndrome characterized by progressive BM failure and cancer susceptibility (14, 15). Since patients with FA uniformly develop BM failure and have high risk of progression to leukemia, it has been long speculated that FA proteins play a specific role in hematopoiesis by governing responses to both genotoxic and cytotoxic stresses (14, 15). Compelling evidence suggests that chronic cytotoxic or genotoxic stresses differentially affect FA hematopoiesis by causing excessive apoptosis of HSPCs and BM niche cells (14, 15). However, whether the FA pathway is involved in the HSC-BM niche crosstalk and in regulating HSC regeneration remains elusive. Our results suggest that FA proteins are required for hematopoietic recovery and HSC regeneration in response to irradiation. These findings add another layer to the current understanding of the role of the FA pathway in regulating hematopoiesis.

HSCs reside in the BM niches, which are specified local tissue microenvironments that promote the maintenance of the stem cells and regulate their function by producing factors that act directly on stem cells (60). Altered mesenchymal niche cells impede generation of normal hematopoietic progenitor cells (61). Different types of stromal cells, including LepR^+^ cells as well as a few types of hematopoietic cells, such as megakaryocytes and macrophages, are the major components of BM niches (62). It has been shown that LepR^+^ MSCs elaborate multiple factors, including stem cell factor and CXCL12, which regulate hematopoiesis (6, 7). Here, we identified LepR^+^CXCL12^+^ niche stromal cells as the source of paracrine Wnt5a that promoted HSC regeneration after irradiation (Figure 4). We showed that irradiation induced significant Wnt5a expression in WT mice but not FA mice (Figure 4 and Supplemental Figure 4). Irradiation also enriched for Wnt5a-expressing BM LepR^+^CXCL12^+^ stromal cells (Supplemental Figure 4). Mechanistically, we showed that FA deficiency leads to reduced levels of both steady-state and radiation-induced c-Myb and Pax2, 2 well-known *Wnt5a* transcription activators, in MSCs from *Fanca^–/–^* and *Fancc^–/–^* mice, and that genetic correction of FA deficiency rescued both steady-state and radiation-induced c-Myb and Pax2. We further showed that FA complementation or forced expression of Pax2 restored *Wnt5a* expression in *Fanca^–/–^* and *Fancc^–/–^* MSCs (Figure 8). We propose that the FA proteins regulate stromal *Wnt5a* expression, possibly through modulating the transcription activator Pax2. Taken together, our present study suggests that FA deficiency impairs Wnt5a production in the BM microenvironment in response to irradiation, which may constitute one cause for impaired radiation-induced regeneration of FA HSCs.

PROX1 plays critical functions in a variety of tissues, including in the lens, heart, liver, pancreas, and CNS (63). Functional inactivation of the *Prox1* gene in mice leads to embryonic lethality (36, 37). However, the role of Prox1 in hematopoiesis, especially under stressed conditions, is less understood. One recent study suggests that Prox1 may function as a potential antagonist of HSC self-renewal (39). Our current study showed that irradiation induced aberrant *Prox1* upregulation in HSCs deficient for the DNA damage repair genes *Fanca* or *Fancc* (Figure 3), leading to loss of HSC quiescence (Supplemental Figure 3, C and D). Lineage-specific deletion of *Prox1* improved long-term repopulation of FA HSCs and aged HSCs after irradiation (Figures 3 and 6). Conversely, forced expression of *Prox1* in WT HSCs mimicked the defective repopulation phenotype of FA HSCs (Figure 3). These results suggest that Prox1 plays a crucial role in the regulation of HSC regeneration during radiation injury and aging.

Wnt signaling plays critical roles in the development, growth, and homeostasis of various organs (16, 17). The binding of Wnt to receptor complexes activates β-catenin–dependent canonical and β-catenin–independent noncanonical signaling pathways (64). It has been shown that noncanonical Wnt5a can inhibit canonical Wnt signaling downstream of β-catenin stabilization through the calcium-dependent activation of Nemo-like kinase (65) as well as other calcium-independent mechanisms (16). Wnt5a competes with Wnt3a for binding to Fzd2, thereby inhibiting β-catenin accumulation and β-catenin–dependent Wnt signaling (66). Recent studies identified Prox1 as a target of β-catenin–TCF/LEF signaling both in vitro and in vivo (29, 44, 45). Consistent with these findings, our mechanistic studies showed that irradiation-induced Wnt5a secretion from LepR^+^ stromal cells inhibited β-catenin accumulation and repressed *Prox1* transcription in HSCs, which subsequently impeded hematologic recovery and HSC regeneration (Figure 5). Furthermore, we observed the detrimental effect of dysregulated Wnt5a/Prox1 signaling on HSC regeneration and hematopoietic recovery in aged mice (Figure 6) and samples from patients with FA (Figure 7). Thus, our results identified a paracrine Wnt5a/Prox1 signaling axis in regulating HSC regeneration under conditions of injury and aging.

## Methods

### Animals and treatment.

*Fanca^+/–^* and *Fancc^+/–^* mice were provided by Madeleine Carreau (Laval University, Quebec City, Quebec, Canada) and Manuel Buchwald (Hospital for Sick Children, University of Toronto, Toronto, Ontario, Canada), respectively (67, 68). Heterozygous *Prox1^fl/+^* mice (42) in a C57BL/6 background were recovered from the sperm purchased from Experimental Animal Division at RIKEN BioResource Center (RIKEN, RBRC10791). The in vitro fertilization (IVF) procedure was performed in the Genetically Engineered Murine Model Core at University of Virginia. Heterozygous *Prox1^fl/+^* mice were interbred with *Vav1-Cre* mice (the Jackson Laboratory; stock 008610) to generate *Prox1^fl/fl^Vav1-Cre* mice and *Prox1^fl/fl^* littermates. *Ctnnb1^fl/fl^* mice (50) were purchased from the Jackson Laboratory (stock 004152) and crossed with *Vav1-Cre* mice to generate *Ctnnb1^fl/fl^Vav1-Cre* mice and *Ctnnb1^fl/fl^* littermates. *Wnt5a^fl/fl^* mice (47) were crossed with *LepR-Cre* mice (the Jackson Laboratory, stock 032457; ref. 13), and then crossed with *Fanca^–/–^* or *Fancc^–/–^* mice to generate *Wnt5a^fl/fl^LepRCre;Fanca^–/–^* (*Wnt5a^–/–^;Fanca^–/–^*) or *Wnt5a^fl/fl^LepRCre;Fancc^–/–^* (*Wnt5a^–/–^;Fancc^–/–^*) offspring.

For aging experiments, both male and female young (10–12-week-old) or aged (20–26-month-old) C57BL/6 mice (69) were used for aging study. All the animals, including BoyJ (C57BL/6: B6, CD45.1^+^) recipient mice, were maintained in the animal facility at UPMC Hillman Cancer Center. For in vivo Wnt5a treatment, the indicated mice were i.p. injected with Wnt5a (50 μg/kg; R&D Systems) every other day for 3 weeks (46).

### Radiation studies.

Eight-week-old WT, *Fanca^–/–^*, or *Fancc^–/–^* mice were irradiated with 500 cGy TBI using a Shepherd cesium 137 irradiator (2). At the indicated time point after TBI, mice were euthanized, and BM cells were isolated for further analysis. Equal numbers of male and female mice from each genotype were used for all studies. PB and BM cells were collected from mice in each group at days +5, +10, or +15 for analysis of hematopoiesis. Complete blood counts were measured using a HemaVet 950 instrument (Drew Scientific). For survival studies, mice were monitored daily through day +30 and euthanized as prescribed by the animal use protocol, if necessary.

### BM transplantation.

First, 100 BM SLAM cells from nonirradiated and irradiated WT, *Fanca^–/–^*, and *Fancc^–/–^* mice or 1000 progeny cells from cocultures, along with 2 × 10^5^ protector cells from congenic BoyJ mice (CD45.1^+^), were transplanted into lethally irradiated (11.75 Gy) BoyJ mice. For serial BMT, 1 to 3 million WBMCs from primary recipients were pooled and injected into sublethally irradiated (7.0 Gy) BoyJ recipients. Donor-derived chimera were detected by flow cytometry at 4, 8, 12, and 16 weeks after transplant using antibodies against CD45.1 and CD45.2.

### Statistics.

GraphPad Prism 9.0 was used for all statistical analysis. All data were checked for normal distribution and similar variance between groups. Data were derived from multiple independent experiments from distinct mice or cell culture plates. Sample sizes for in vitro studies were chosen based on observed effect sizes and standard errors from prior studies. For all animal studies, a power test was used to determine the sample size needed to observe a 2-fold difference in means between groups with 0.8 power using a 2-tailed Student’s *t* test. All animal studies were performed using sex- and age-matched animals with WT littermates as controls. Animal studies were performed without blinding of the investigator, and no animals were excluded from the analysis. All comparisons performed were done using a 2-tailed Student’s *t* test, unless otherwise indicated in the figure legends. Values are reported as mean ± SD, unless stated otherwise. Paired or unpaired Student’s *t* test was used for 2-group comparison, and 1-way ANOVA was used for comparison of more than 2-groups. *P* values less than 0.05 were considered statistically significant.

### Study approval.

All experimental procedures conducted in this study were approved by the IACUC of University of Pittsburgh. Primary human samples were obtained after written informed consent at Cincinnati Children’s Hospital Medical Center Respiration Core or Pittsburgh Biospecimen Core at University of Pittsburgh under the approved IRB protocols.

For detailed experimental procedures, see Supplemental Methods.

## Author contributions

QL and LW performed the research and analyzed the data; SC, FAC, NA, and JJ performed some of the research and assisted with data analysis. WD designed the research, analyzed the data, and wrote the paper.

## Supplementary Material

Supplemental data

## Version 1 06/15/2022

Electronic publication
