# Supplementary material for: Hematopoietic stem cell regeneration through paracrine regulation of the Wnt5a/Prox1 signaling axis
Source: J Clin Invest. 2022 Jun 15;132(12):e155914. doi: 10.1172/JCI155914 (PMC9197516; doi:10.1172/JCI155914)
Supplement: Supplemental data [file jci-132-155914-s087.pdf]

Supplementary materials

Histopathology and immunofluorescence staining

Hematoxylin and eosin (H&E) staining of femurs from the mice was performed as previously described (1, 2). Femurs were fixed overnight in 4% paraformaldehyde, decalcified and paraffin-embedded. Sections were then stained with Hematoxylin and Eosin staining solution (H&E solution; Sigma-Aldrich, St Louis, MO) and examined at X20 magnification by microscope.

Flow cytometry analysis

The lineage marker (Lin) mixture (Cat # 559971, BD BioSciences, San Jose, CA) for BM cells from treated or untreated mice includes the following biotinylated antibodies: CD3ε (145-2C11), CD11b (M1/70), CD45R/B220 (RA3-6B2), mouse erythroid cells Ly-76 (Ter119), Ly6G and Ly-6C (RB6-8C5). Other conjugated antibodies used for surface staining include: CD45.1 (A20; Cat # 553776), CD45.2 (A104; Cat # 553772), Sca1 (D7; Cat # 560654), c-kit (2B8; Cat # 560185), CD48 (HM48-1; Cat # 560731), CD150 (9D1; Cat # 562647), CD11b (Mac1; Cat # 561015) and CD3ε (Cat # 553067) (all from BD Biosciences, San Jose, CA); Gr1 (Cat # 47-5931-82) and B220 (Cat #: 12-0452-82; both from Invitrogen, Grand Island, NY), and VE-Cadherin (AF-647, Cat # 138006; BioLegend, San Diego CA). Biotinylated primary antibodies, including LepR antibody (Cat # BAF497; R&D System, Minneapolis, MN) were detected by incubation of antibody coated cells with streptavidin-PerCP or FITC (Cat # 551419 and 554060; BD Biosciences, San Jose, CA) in a two-step staining procedure. For some of the experiments, pacific blue conjugated CD45.2 (A104, Cat # 368539; BioLegend, San Diego, CA) was used to determine donor-derived cells.

For intracellular flow cytometry analysis, cells were fixed and permeabilized using BD Cytofix/Cytoperm buffer (Cat # 554722; BD BioSciences, San Jose, CA) and subsequently incubated with primary antibodies against β-catenin (Alexa 647-conjugated, Cat # 4627S; Cell Signaling, Danvers, MA), Wnt5a (3D10; Cat # MA5-15511; ThermoFisher, Waltham, MA) or CXCL12 (; Cat # 578702; BioLegend, San Diego CA). IgG isotype controls (eBioscience, Waltham, MA) were used for each fluorophore (3).

For cell cycle analysis, surface marker-stained cells were fixed and permeabilized using Cytofix/Cytoperm buffer (BD Pharmingen, Cat # 554722) followed by intensive wash using Perm/Wash Buffer (BD Pharmingen, Cat # 554723). Cells were then labeled with Ki67 (Invitrogen, Cat # 11-5698-82) and DAPI (Sigma-Aldrich, Cat # CAS28718-90-3) at 37˚C for 1 hour followed by flow cytometry analysis. $CD45.2^{+}$SLAM cells were gated for analysis.

For apoptosis staining, surface marker-stained cells were incubated with Annexin V and 7AAD using the BD ApoAlert Annexin V Kit (BD Pharmingen, San Jose, CA) in accordance with the manufacturer's instruction. Flow cytometry analysis was then performed to determine the proportion of Annexin V-positive cells. $CD45.2^{+}$SLAM cells were gated for analysis.

Colony-forming unit assay

Low density bone marrow cells (LDBMCs) isolated from the indicated mice were plated in a 35-mm tissue culture dish in 4 mL of semisolid medium containing 3 mL of MethoCult M3134 (Cat # 03134; Stem Cell Technologies, Vancouver, BC, Canada) and the following growth factors: 100 ng/ml SCF (Cat #: 250-03), 10 ng/ml IL-3 (Cat # 213-13), 100 ng/ml

GM-CSF (Cat # 315-03) (all from Peprotech, Burlington, NC), and 4 units/mL erythropoietin (Cat # 587602; BioLegend, San Diego CA). On day 7 after plating, erythroid and myeloid colonies were enumerated. Hematopoietic clonal growth results were expressed as means (of triplicate plates) ± SD of three experiments.

Isolation and culture of LepR$^+$ bone marrow cells

BM cells from femurs and tibias was isolated by crushing bones and then digesting them for 10 min at 37 ºC in collagenase and dispase (Cat # 10 269 638 001; Sigma-Aldrich, St Louis, MO). Cells were then rinsed and depleted of lineage-committed cells with the Miltenyi lineage-depletion kit (Cat # 130-090-858; Miltenyi Biotec, San Diego, CA). The linage-negative fraction was stained with two antibodies for Leptin receptor: LepR-PE (Cat # LS-C261834-200; Lifespan Bioscience, Seattle, WA) and LepR-FITC (Cat #: LS-bs-0109R-FITC; Bioss Antibodies, Woburn, MA) in addition to CD45 (Cat #: 553081) and 7AAD (Cat # 559925; BD PharMingen, San Jose, CA). Cells were sorted on a Sony MA900 cell sorter (Sony BioTech, San Jose, CA) for 7AAD$^-$CD45$^-$LepR$^+$ subset.

For LepR$^+$ cell culture, sorted cells were seeded at a density 10 cells/cm^2 in 12-well plates in DMEM (Cat # 10565018; Gibco, Fort Worth, TX) supplemented with 20% FBS (Sigma-Aldrich, St Louis, MO), 10 mM Y-27632 (Cat # 1254; TOCRIS, Minneapolis, MN) and 1% penicillin/streptomycin (Cat # 10378016; Invitrogen, Grand Island, NY). The cultures were incubated at 37 ºC in a humidified atmosphere with 5% O_2 and 10 % CO_2 for 7-10 days (4). For Wnt5a treatment, rWnt5a (Cat # 645-WN-010/CF; 100 ng/ml; R&D System, Minneapolis, MN; 5) was added to the co-culture of 70-80% confluent LepR$^+$ cells and WT LSK or SLAM cells. For Wnt5a neutralization, anti-Wnt5a (Cat # AF645; 2 μg/ml; R&D Systems, Minneapolis, MN; 6) was added to the co-culture.

RNA Isolation, Reverse Transcriptase (RT)-PCR

Total RNA was extracted from the indicated cells of each mouse genotype and treated with RNase-free DNase to remove contaminating genomic DNA. Reverse transcription was performed with random hexamers and Superscript II RT (Cat # 18064022; Invitrogen, Grand Island, NY) and was carried out at 42 °C for 60 min and stopped at 95 °C for 5 min. First-strand cDNA was used for real-time PCR using primers listed in Table S1. Samples were normalized to the level of *GAPDH* mRNA.

RT^2 profiler PCR Wnt Array

SLAM or $CD45^-LepR^+$ cells were isolated from the indicated mice. Total RNA was extracted suing the TRIZOL reagent and further purified using the miRNeasy Mini Kit (Cat # 217084; QIAGEN, Germantown, MD). RNA (0.5 μg) was analyzed by the reverse transcription (RT^2) profiler polymerase chain reaction array mouse Wnt signaling pathway array according to the manufacturer's protocol. The cycle threshold values were defined automatically by the CFX manager software (BioRad Laboratories, Hercules, CA) as the fractional cycle number at which the fluorescence passes an arbitrarily set threshold. Analysis of the data was done using Microsoft Excel.

ELISA

Whole BM from one femur was collected from the indicated mice 24h post 500 cGy TBI. After centrifugation, BM supernatants were collected into Isove's modified Dulbecco's medium (IMDM) and analyzed for Wnt5a concentration using Wnt5a ELISA kit (Cat# CSB-EL026138MO; CUSABIO, Houston, TX) following the manufacturer's instruction.

Molecular cloning and materials

To generate lentiviral expression vectors, the *Prox1* cDNA (purchased from Origene, Rockville, MD; Cat # MC205549) was cloned into the pLVX-IRES-GFP vector (Cat # 128652; Addgene, Watertown, MA) as previously described (7).

Lentivirus was produced in 293 T cells after transfection of 20 µg plasmid, 15 µg pCMVΔ8.91 helper plasmid and 6 µg pMD.G using standard calcium phosphate transfection procedures. Fresh medium change was performed 12 hours after transfection. Supernatants from the cell culture were collected 48 hours after transfection, filtered through 0.45 µm-pore-size filters, and concentrated at 25,000 rpm for 2.5 hours at 4°C to harvest viral particles. Virus pellet was resuspended in sterile PBS and stored at -80°C.

Lentiviral transduction was performed as previously described (8). Briefly, sorted BM LSK cells were pre-stimulated for 5-10 hr in a 24-well dish in serum-free medium. The lentiviral media were added to the cells, spinoculated for 90 min at 270G in the presence of 8 µg/ml polybrene (Cat # TR-1003; Sigma-Aldrich, St Louis, MO). This process was repeated 24 hr later with a fresh batch of lentiviral media.

To generate retroviral expression vectors, the *c-Myb or Pax2* cDNA (purchased from Origene, Rockville, MD; Cat # MR209649; MR227386) were cloned into MIEG3-eGFP vector (9). To complement FA deficiency, MSCs from WT, *Fanca*$^{-/-}$ and *Fancc*$^{-/-}$ mice were transduced with retroviral vector expressing eGFP, eGFP-FANCA or eGFP-FANCC (9).

Retroviral production and transduction of MSCs was performed as previously described (10, 11). Briefly, recombinant retroviruses were produced using the Phoenix

cell packaging system by transient expression of relevant cDNAs in the MIEG3 retroviral vector containing bicistronically expressed eGFP (10, 11). The indicated cells were infected with the respective retroviruses and harvested 48 h post-infection. eGFP-positive cells were used for different assays, as indicated in Results.

Human bone marrow stromal cell (hBMSC) culture and co-culture

All studies with human material were approved by the institutional review board and ethics committee of the University of Pittsburgh. Written informed consent was obtained from all subjects. Human MSC culture protocol was adapted and modified from a previously described method (12, 13). Human MSC cultures were maintained at 37 ºC in 5% CO2 and 98% humidity in normal growth medium consisting of Dulbecco's modified eagle medium (DMEM-low glucose, with GlutaMAX; ThermoFisher Scientific, Reinach, Switzerland), supplemented with 10% fetal bovine serum (FBS; Sigma-Aldrich, St Louis, MO, USA), penicillin/streptomycin (50 units/ml; 50 lg/ml). All MSCs were used at passage 3.

BM $CD34^+$ cells from healthy donors (HD) were seeded on 70% confluent MSCs derived from the indicated donors and cultured in serum-free medium containing 100 ng/ml each of SCF, FLT3 ligand, thrombopoietin (TPO, also term THPO) and granulocyte colony-stimulating factor (G-CSF) (all from Peprotech, Cat #: 30007, 30019, 30018, 30023, Rocky Hill, NJ). Cells were maintained at 37 °C in a humidified atmosphere containing 5% CO2. The co-cultures were incubated for 5 days, at which time-periods the progeny of the co-cultured HSPCs was harvested and analyzed (13).

Western blotting

Whole cell lysates (WCL) were extracted from the indicated cells using 1X cell lysis RIPA buffer (Cat # 89900; ThermFisher, Waltham, MA) supplemented with a protease inhibitor cocktail (Thermo Scientific, Waltham, MA) and PMSF. Western blotting was performed as described previously (14). Anti-c-Fos (Cat #: sc-166940; Santa Cruz), anti-c-Myb (Cat #: NBP2-21659; Novus), anti-CutL1 (Cat #: NBP3-10866; Novus), anti-DLX5 (Cat #: NBP1-85793; Novus), anti-SP-1 (cat # NB600-232; Novus), anti-Pax2 (cat # H00005076-M01; Novus), anti-FOXG1 (Cat #: ab18259; Abcam) antibodies were used for western blotting analysis. Signals were visualized by incubation with anti-mouse or anti-rabbit secondary antibody, followed by enhanced chemiluminescence (Amersham Biosciences, Pittsburgh, PA). Western blot images were taken using Amersham Imager 680 chemiluminescence imaging system (Amersham Biosciences, Pittsburgh, PA) for both colorimetric markers and chemiluminescence images. Quantification of the obtained western blots was performed by densitometry on ImageJ 1.8.0 (Bethesda, MD).

References:

1. Doan PL, et al. Tie2^{+} bone marrow endothelia cells regulate hematopoietic stem cell regeneration following radiation injury. *Stem Cells* 2013;31:327-337.
2. Ma Z, et al. Hes1 deficiency causes hematopoietic stem cell exhaustion. *Stem Cells.* 2020;38(6):756-768.
3. Cohen SB, et al. β-Catenin Signaling Drives Differentiation and Proinflammatory Function of IRF8-Dependent Dendritic Cells. *J. Immunol.* 2015;194(1):210-222.
4. Zhou BO, et al. Leptin-receptor-expressing mesenchymal stromal cells represent the main source of bone formed by adult bone marrow. *Cell Stem Cell.* 2014;15:154-168.
5. Florian MC, et al. A canonical to non-canonical Wnt signaling switch in haematopoietic stem-cell ageing. *Nature.* 2013;503(7476):392-396.
6. Hasan MK, et al. Wnt5a induces ROR1 to complex with HS1 to enhance migration of chronic lymphocytic leukemia cells. *Leukemia.* 2017;31:2615-2622.
7. Lin Q, et al. Persistent DNA damage-induced NLRP12 improves hematopoietic stem cell function. *JCI Insight.* 2020;5(10):e133365.

8. Zhao H, et al. Amelioration of murine beta-thalassemia through drug selection of hematopoietic stem cells transduced with a lentiviral vector encoding both gamma-globin and the MGMT drug-resistance gene. *Blood.* 2009;113(23):5747-5756.
9. Zhang X, et al. Defective homing is associated with altered Cdc42 activity in cells from patients with Fanconi anemia group A. *Blood.* 2008;112(5):1683-1686.
10. Rani R, Li J, Pang Q. Differential p53 Engagement in Response to Oxidative and Oncogenic Stresses in Fanconi Anemia Mice. *Cancer Res.* 2008;68(23):9693-9702.
11. Du W, et al. A cytoplasmic FANCA-FANCC complex interacts and stabilizes the leukemic NPMc protein. J Biol Chem. 2010;285(48):37436-37444.
12. Wu L, et al. Leukemia mesenchymal stromal cell COX2-PG signaling regulates donor anti-leukemia immunity. *Br J Haematol.* 2018;183(3):445-456.
13. Wu L, et al. Mesenchymal COX2-derived PGD2 activates an immunosuppressive ILC2-Treg axis and promotes leukemic cell proliferation. *Leukemia.* 2020;34(11):3028-3041.
14. Wu L, et al. Quinacrine-CASIN combination overcomes cytarabine resistance in human acute lymphoid leukemia. *Nat Commun.* 2021;12(1):6936.

Supplementary tables

Table S 1. Primers used for qPCR analysis

Gene	Forward	Reverse
Atm	ATC CCT TGT GTG TTC TCT G	CGC CTC TGC TGT CGT GTA T
Atr	CACTGAATGATGAATGTGGGATTAT	ATGAGTCAGTCGAAATGGTACA
Brca1	CGAGGAAATGGCAACTTGCCTAG	TCACTCTGCGAGCAGTCTTCAG
Eepd1	AGGCTCGAAGTCTCTGGACA	GCATCCTTCTTGCTCCAGTC
Eya2	TCGGGACGACTCTGCACTGT	GCTGTACTGTGTCTGGCCATAGG
Gadd45b	GTTCTGCTGCGACAATGACA	TTGGCTTTTCCAGGAATCTG
Prkdc	GAATTGCATTAGTGCTGTGGTGCAC	GTACGTAGGTACTTTCCACCTGC
Prox1	TGAAGACCTACTTCTCGGAGG	TGCTGAACCACTTGATGAGCT
Rad51c	GTCTCTGAGGCAGGGTCTTC	TCAATTCTCAGAGCCCACAG
Trp53i13	ACTGGAGCTGGCATGGATA	ACAGCTTCCCTCTTCTGGAG
Wnt5a	GGAACGAATCCACGCTAAGGGT	AGCACGTCTTGAGGCTACAGGA
Xrcc5	GAGGACACTATTCAAGGGTACCG	GCAACAGCTGCCGCCTCATCATC
Pax2	ATTCCTCGCTCCAACGGTGAGA	CAGACCAGATGTAAACCTCCACC
Gapdh	TCAATGAAGGGGTCGTTGAT	CGTCCCGTAGACAAAATGGT
WNT5a	TACGAGAGTGCTCGCATCCTCA	TGTCTTCAGGCTACATGAGCCG
PROX1	TCACCTTATTCGGGAAGTGC	GTACTGGTGACCCCATCGTT
GAPDH	GTCTCCTCTGACTTCAACAGCG	ACCACCCTGTTGCTGTAGCCAA

Supplementary figures

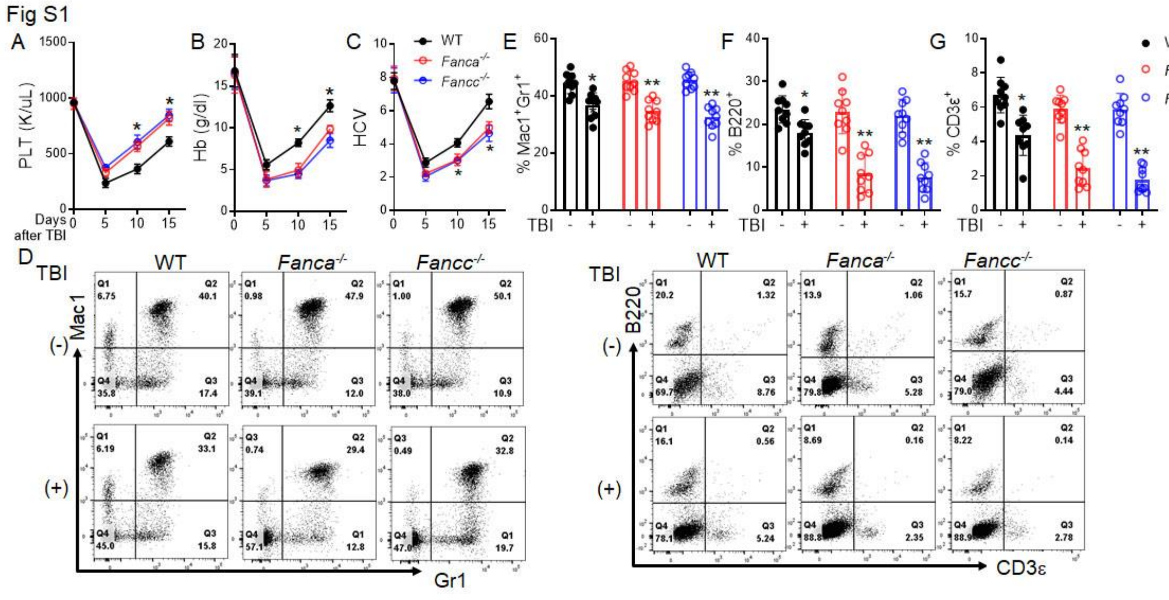

Fig S1. FA deficiency compromises hematopoietic recovery following irradiation. (A-C) PB platelet (PLT; A), Hb (B) and HCV (C) in WT, *Fanca*$^{-/-}$ or *Fancc*$^{-/-}$ mice at the indicated timepoints after 500 cGy TBI (n= 12/group). (D-G) Representative flow plots (D) and quantification of Myeloid (Mac1/Gr1, E), B cell (B220, F) and T cell (CD3ε, G) in the BM of WT, *Fanca*$^{-/-}$ or *Fancc*$^{-/-}$ mice on day +15 following 500 cGy TBI (n = 9).

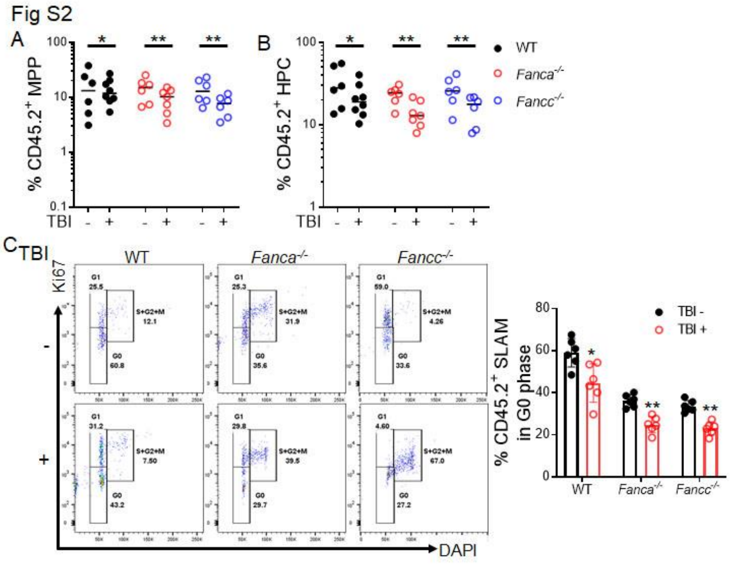

Fig S2. FA deficiency dampens HSC regeneration following irradiation. (A, B) Mean percentages of donor CD45.2$^+$ MPP (LSKCD48$^+$CD150$^+$, A) and HPC (LSKCD48$^-$CD150$^-$, B) cells in the BM of CD45.1$^+$ recipient mice at 16 weeks following competitive transplantation of BM cells collected from the indicated mice at day +15 following 500 cGy TBI (n = 6-8). (C) Cell cycle analysis of donor-derived HSCs in the recipients transplanted with irradiated WT, *Fanca*$^{-/-}$ or *Fancc*$^{-/-}$ cells. CD45.2$^+$ SLAM cells were gated for analysis. Representative flow plots (Left) and quantifications (right) are shown (n= 6).

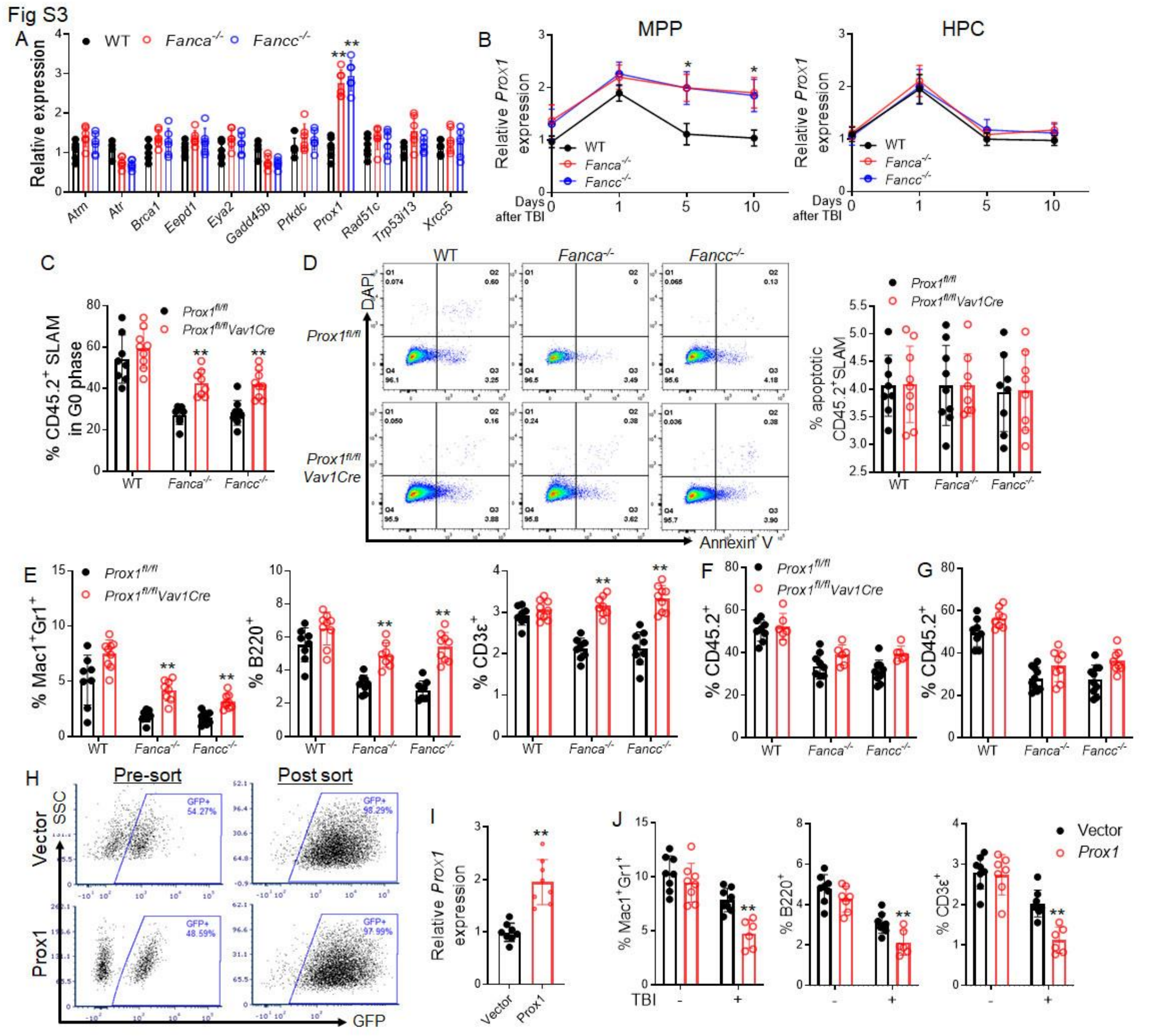

Fig S3. Prox1 regulates HSC regeneration following irradiation. (A) qPCR analysis

of the indicated DDR genes in BM HSCs from WT, *Fanca*$^{-/-}$ or *Fancc*$^{-/-}$ mice at 24h following 500 cGy TBI. Samples were normalized to the level of *GAPDH* mRNA of non-irradiated HSCs. Relative mRNA expression levels were normalized to that of non-irradiated WT mice (n = 6). (B) Base-line and irradiation-induced *Prox1* expression in BM MPP and HPCs of the non-irradiated mice and irradiated mice at day +1, 5, 10 (n = 6). (C) *Prox1* deletion improves HSC quiescence. LSK (Lin$^-$Sca1$^+$c-kit$^+$) cells from the indicated mice were subjected to 300 cGy irradiation followed by transplantation, along with 2 × 10^5 competing CD45.1$^+$ WT BM cells, into lethally irradiated BoyJ recipients. Cell cycle analysis were conducted at 16 weeks post BMT. CD45.2$^+$ SLAM cells were gated for analysis (n= 8-9). (D) *Prox1* deletion does not affect HSC apoptosis. Annexin V/DAPI staining of donor SLAM cells of recipients described in (C). (E) Mean percentages of donor Myeloid (Mac1/Gr1), B cell (B220) and T cell (CD3ε) in the BM of the mice described in Fig 3C at 16 weeks post BMT (n = 8-9). (F) Donor-derived chimera in the primary recipients transplanted with cells from non-irradiated mice. LSK cells from non-irradiated control mice for Fig 3C were transplanted, along with 2 × 10^5 competing CD45.1$^+$ WT BM cells, into lethally irradiated BoyJ recipients. Donor-derived chimera were determined at 16 weeks post BMT (n = 6-9). (G) Donor-derived chimera in the secondary transplanted recipients transplanted with cells from non-irradiated mice. WBMCs from recipients described in (C) were injected into sublethally irradiated BoyJ recipients. Donor-derived chimera were determined 16 weeks post BMT (n =8-10). (H) Flow cytometry analysis of WT LSK cells transduced with lentiviral vector expressing GFP or GFP-Prox1. Flow plots of pre-sorting and post-sorting are shown. (I) qPCR of *Prox1* expression in sorted transduced cells (n= 8 assays). (J) Mean percentages of donor

Myeloid (Mac1/Gr1), B cell (B220) and T cell (CD3ε) in the BM of the primary recipients transplanted with cells described in Fig 3F at 16 weeks post BMT (n = 6-8).

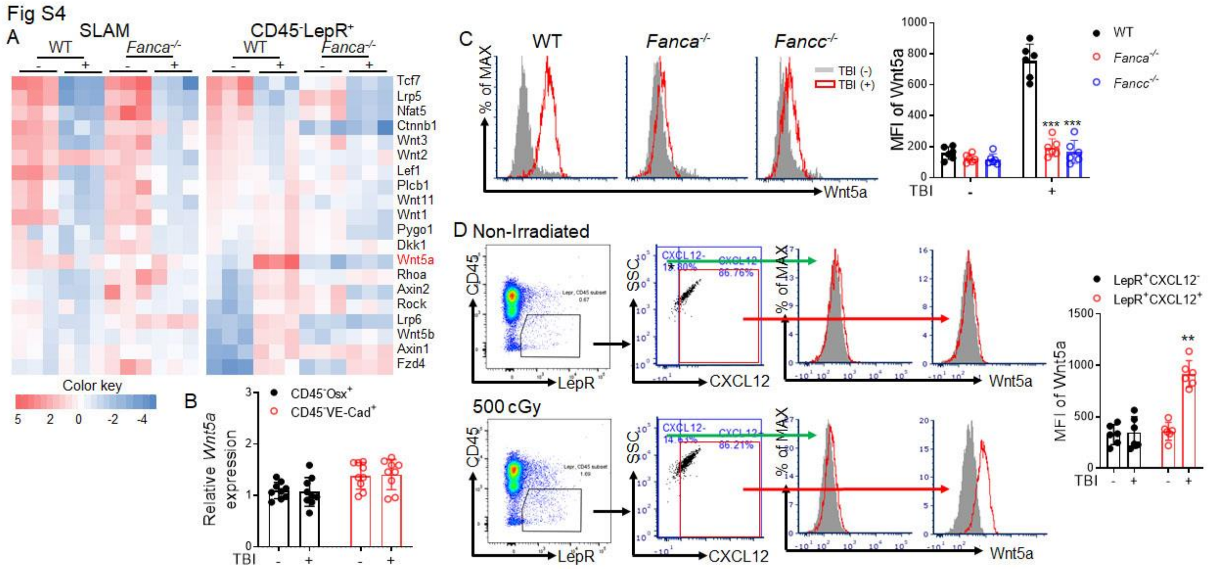

Fig S4. Irradiation induces Wnt5a expression in LepR^{+}CXCL12^{+} cells of WT mice. (A) Heatmap of Wnt pathway gene expression profile in BM SLAM cells (Left) and BM CD45^{-}LepR^{+} cells (Right) from non-irradiated and irradiated WT and *Fanca*$^{-/-}$ mice at 48 h following 500 cGy TBI (n = 3 assays). (B) *Wnt5a* expression by qPCR in FACS-sorted CD45^{-}Osx^{+} and CD45^{-}VE-Cad^{+} cells from non-irradiated mice and irradiated mice at 24 h following 500 cGy TBI (n = 9). (C) Left: Representative flow cytometry analysis of intracellular Wnt5a protein in BM CD45^{-}LepR^{+} stromal cells from non-irradiated and irradiated mice at 24 h following 500 cGy TBI. Right: Quantification of intracellular Wnt5a protein by flow cytometry analysis (n = 6). (D) Irradiation enriches for Wnt5a-expressing BM LepR^{+}CXCL12^{+} stromal cells in WT mice. Left: representative flow of Wnt5a expression in LepR^{+}CXCL12^{+} and LepR^{+}CXCL12^{-} subsets from non-irradiated and irradiated mice at 24 h following 500 cGy TBI. Right: mean percentages of BM

Wnt5a$^+$LepR$^+$CXCL12$^+$ cells and Wnt5a$^+$LepR$^+$CXCL12$^-$ cells from non-irradiated and irradiated mice at 24 h following 500 cGy TBI (n = 6).

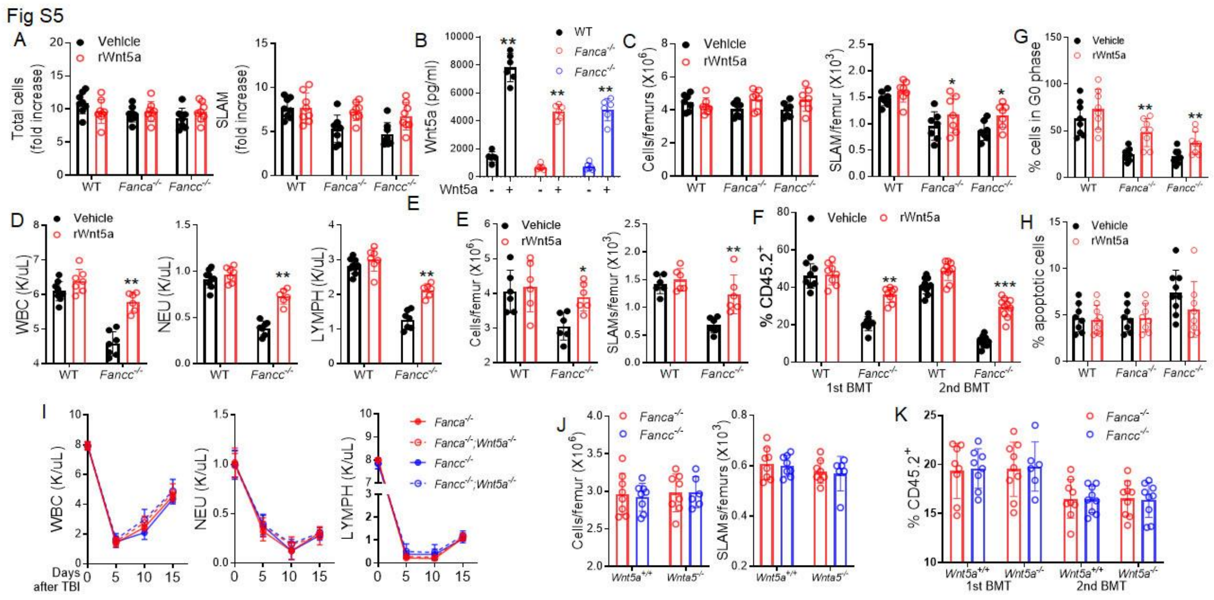

Fig S5. Wnt5a from LepR$^+$CXCL12$^+$ cells regulates hematopoietic recovery and HSC regeneration following irradiation. (A) Fold increase of total cell numbers (left) and SLAM cells (right) after 5 days of culture of non-irradiated BM SLAM cells in medium supplemented with cytokines (thrombopoietin 20 ng/mL, SCF 100 ng/mL, Flt-3 ligand 50 ng/mL) and rWnt5a (100 ng/ml) or vehicle (saline). (n = 8). (B) rWnt5a levels in the BM of mice at 1 h following administration of rWnt5a (50 μg/kg) or vehicle (saline) (n = 6). (C) Mean total BM cell numbers (left) and SLAM cells (right) after 3 weeks of systemic treatment with rWnt5a (50 μg/kg) in non-irradiated mice (n = 7). (D) PB WBC, NEU and LYMPH counts in WT and *Fancc*$^{-/-}$ mice at day 22 post-TBI. Mice were subjected to 500 cGy TBI and treated with rWnt5a (50 μg/kg; i.p.) or vehicle (saline) every other day for 3 weeks (n =6-9). (E) Total BM cells (left) and SLAM cells (right) in WT and *Fancc*$^{-/-}$ mice on day 22 after 500 cGy TBI and treatment with rWnt5a or vehicle (saline) (n = 6). (F) 1st (left) and 2nd (right) competitive BMT with cells from the mice in S4H (n= 8-10) (1st BMT,

n=8; 2nd BMT, n=10). (G) Wnt5a treatment improves HSC quiescence. Donor cells from primary recipient mice described in (F) were subjected to cell cycle analysis using Ki67 and DAPI. CD45.2$^+$SLAM cells were gated for analysis. (H) Wnt5a treatment does not affect apoptosis. Donor cells from primary recipient mice described in F were subjected to apoptosis analysis using Annexin V and DAPI. CD45.2$^+$SLAM cells were gated for analysis. (I) PB WBC, NEU, LYMPH counts in *Fanca-Wnt5a-KO* and *Fancc-Wnt5a-KO* mice over time (0, 5, 10, 15 days) following 500 cGy TBI (n = 10). (J) Total BM cells (Left) and SLAM (Right) in *Fanca-Wnt5a-KO* and *Fancc-Wnt5a-KO* mice at day15 following 500 cGy TBI (n = 6-8). (K) 1st (left) and 2nd (right) competitive BMT with cells from the mice in S4K (n = 6-9).

Fig S6

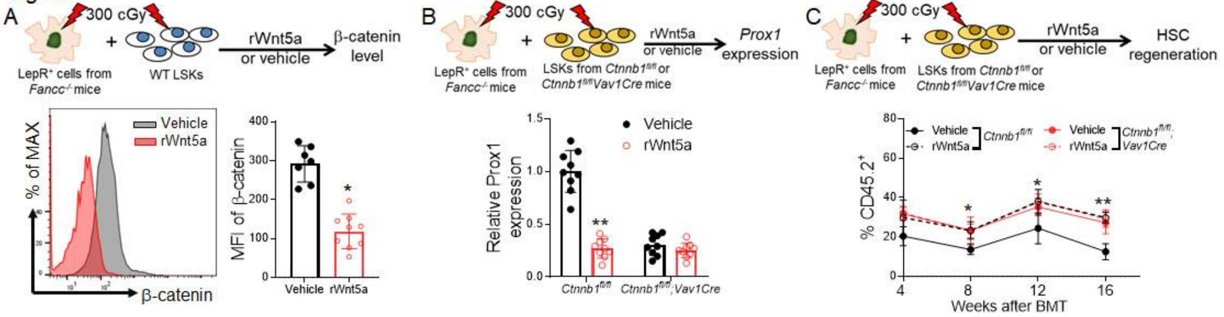

Fig S6. LepR$^+$ niche cell-derived Wnt5a inhibits β-catenin accumulation and represses Prox1 expression in irradiated HSPCs. (A) Wnt5a treatment reduces β-catenin accumulation in co-cultured HSPCs. WT LSK cells and LepR$^+$ cells isolated from *Fancc$^{-/-}$* mice were subjected to 300 cGy irradiation and then co-culture in the presence of rWnt5a (100 ng/ml) or vehicle (saline) for 5 days. β-catenin levels were determined in the suspension cells by flow cytometry analysis (n = 7-9). (B) Deletion of *Ctnnb1* abrogates the effect of rWnt5a on *Prox1* expression in co-cultured HSPCs. LSK cells from *Ctnnb1$^{fl/fl}$* or *Ctnnb1$^{fl/fl}$Vav1Cre* mice and LepR$^+$ cells isolated from *Fancc$^{-/-}$* mice

were subjected to 300 cGy irradiation and then co-culture in the presence of rWnt5a (100 ng/ml) or vehicle (saline) for 5 days. *Prox1* expression was determined by qPCR. Samples were normalized to the level of *GAPDH* mRNA (n = 9). (C) *Ctnbb1* deletion abolishes the promoting effect of rWnt5a on hematopoietic regeneration. 1000 progeny cells from the co-cultures of *Ctnnb1*$^{fl/fl}$ or *Ctnbb1*$^{fl/fl}$*Vav1Cre* LSK cells on *Fancc*$^{-/-}$ LepR^{+} cells in the presence of 100 ng/ml rWnt5a or vehicle were transplanted, along with 2 × 10^5 competing CD45.1^{+} WT BM cells, transplanted into lethally irradiated BoyJ recipients. Donor-derived chimera were detected at the indicated timepoints post BMT (n =10-12).

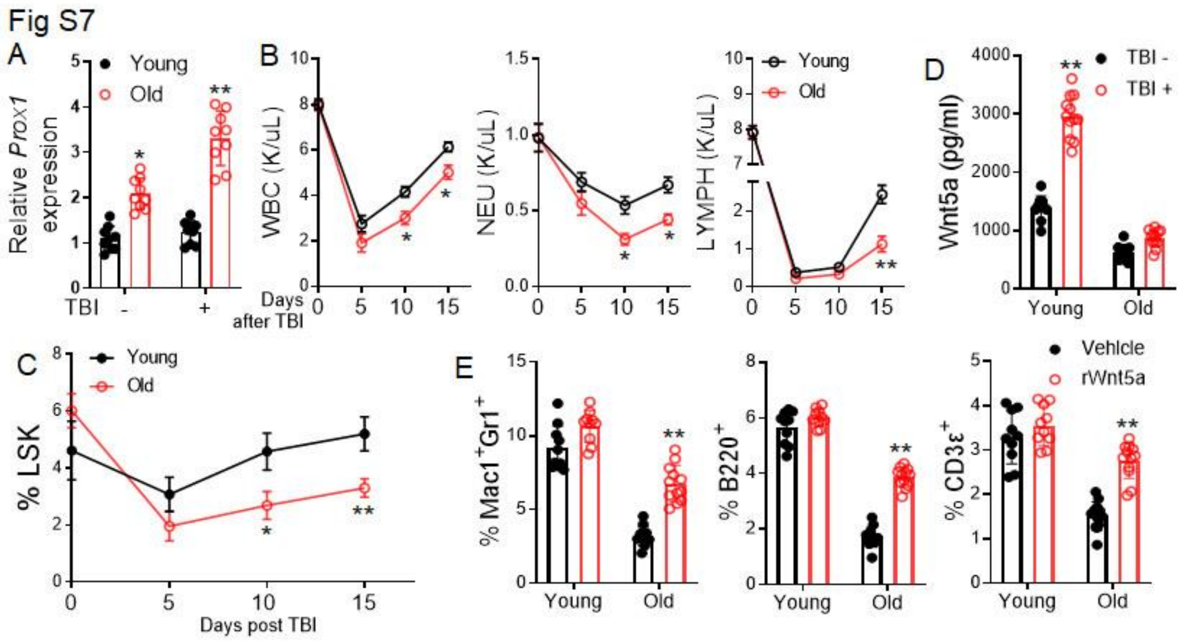

Fig S7. Altering Wnt5a-Prox1 signaling compromises HSC regeneration and hematopoietic recovery in aged mice. (A) Relative *Prox1* expression in non-irradiated and irradiated young and old mice. RNA extracted from SLAM cells isolated from young and old at 24h after 500 cGy TBI was subjected to qPCR analysis using primers listed in Table S1. Samples were normalized to the level of *GAPDH* mRNA. *Prox1* mRNA levels

were normalized to that of non-irradiated young mice (n = 9). (B) PB WBC, NEU, and LYMPPH counts in young and old mice at the indicated timepoints following 500 cGy TBI (n = 8-10). (C) Mean numbers of BM LSK cells in young and old mice at the indicated timepoints following 500 cGy TBI (n = 6). (D) Wnt5a protein levels in the BM supernatants of non-irradiated and irradiated young and old mice at 24h after 500 cGy TBI (n = 6-9). (E) Mean levels of donor lineage cell engraftment at 16 weeks following competitive transplantation in the recipient mice described in Fig 6D (n = 10-12).

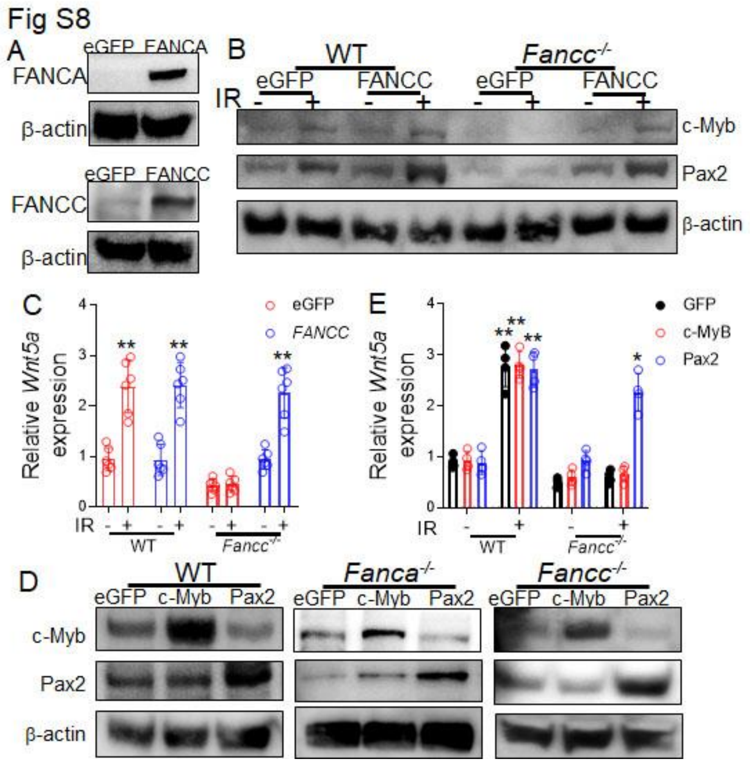

Fig S8. Genetic complementation of FA deficiency. (A) MSCs from *Fanca*$^{-/-}$ or *Fancc*$^{-/-}$ mice were transduced with retrovirus expressing eGFP, eGFP-FANCA or eGFP-FANCC. GFP^{+} cells were sorted 48 hours after transduction followed by whole cell lysate (WCL) extraction and immunoblotting analysis using antibodies against FANCA (Left), FANCC (Right) or β-actin. (B) Genetic correction of *FANCC* deficiency restores both

steady and irradiation-responsive levels of c-Myb and Pax2. MSCs from WT or *Fancc*$^{-/-}$ mice were transduced with retrovirus expressing eGFP or eGFP-FANCC. Sorted GFP^{+} cells were subjected to irradiation (300 cGy). WCL from irradiated or control MSCs were subjected to immunoblotting using antibodies against c-Myb, Pax2 or β-actin. (C) Genetic correction of *FANCC* deficiency rescues both steady and irradiation-induced *Wnt5a* expression in FA MSCs. The MSCs described in (B) were subjected to qPCR analysis for *Wnt5a* expression. Statistics were performed in the indicated groups: Two-tailed, paired *t* test (parametric). ** $p<0.01$. WT + eGFP IR (-) vs WT + eGFP IR (+): $p=0.0052$; WT + FANCC IR (-) vs WT + FANCC IR (+): p=0.0021; *Fancc*$^{-/-}$ + FANCC IR (-) vs *Fancc*$^{-/-}$ + FANCC IR (+): p=0.0045. (D) MSCs from WT (Left), *Fanca*$^{-/-}$ (Middle) or *Fancc*$^{-/-}$ (Right) mice were transduced with retrovirus expressing eGFP, eGFP-c-Myb or eGFP-Pax2. GFP^{+} cells were sorted 48 hours after transfection followed by whole cell lysate extraction, and immunoblotting analysis using antibodies against c-Myb, Pax2 or β-actin. (E) Forced expression of Pax2 restores *Wnt5a* expression in *Fancc*$^{-/-}$ MSCs. MSCs from WT and *Fancc*$^{-/-}$ mice were transduced with lentiviral vector expressing eGFP, c-Myb or Pax2. Sorted GFP^{+} cells were subjected to qPCR analysis for *Wnt5a* expression. Statistics were performed in the indicated groups: Two-tailed, paired *t* test (parametric). ** $p<0.01$. WT + eGFP IR (-) vs WT + eGFP IR (+): $p=0.0053$; WT + c-MyB IR (-) vs WT + c-MyB IR (+): p=0.0028; WT + Pax2 IR (-) vs WT + Pax2 IR (+): p=0.0063; *Fancc*$^{-/-}$ + Pax2 IR (-) vs *Fancc*$^{-/-}$ + Pax2 IR (+): p=0.0175.
